# Emergency Department Visits for Minor Illnesses Among Recent Refugee and Immigrant Children

**DOI:** 10.1001/jamanetworkopen.2025.60070

**Published:** 2026-02-27

**Authors:** Susitha Wanigaratne, Julia Brandenberger, Hong Lu, Therese A. Stukel, Tomi Odugbemi, Rick Glazier, Jen Rayner, Astrid Guttmann

**Affiliations:** 1 Edwin S.H. Leong Centre for Healthy Children, University of Toronto & The Hospital for Sick Children, Toronto, Canada; 2 ICES, Toronto, Canada; 3 Dalla Lana School of Public Health, University of Toronto, Toronto, Canada; 4 Division of Paediatric Emergency Medicine, Department of Paediatrics, Inselspital, Bern University Hospital, University of Bern, Bern, Switzerland; 5 Institute of Health, Policy, Management & Evaluation, University of Toronto, Toronto, Canada; 6 Temerty Faculty of Medicine, University of Toronto, Toronto, Canada; 7 Department of Family and Community Medicine, University of Toronto, Toronto, Canada; 8 Department of Family and Community Medicine, St. Michael’s Hospital, Toronto, Canada; 9 MAP Centre for Urban Health Solutions, St. Michael’s Hospital, Toronto, Canada; 10 Alliance for Healthier Communities, Toronto, Ontario, Canada; 11 Division of Paediatric Medicine, the Hospital for Sick Children, Toronto, Canada; 12 Department of Paediatrics, University of Toronto, Toronto, Canada

## Abstract

**Importance:**

Health care resource constraints across North America are leading to decreased access to primary care, particularly for newcomers. In Canada, several pathways to permanent residency shape early navigation to the publicly funded health care system.

**Objective:**

To explore access to primary care by analyzing the percentage of all minor illness visits seen in an emergency department (ED) in recently arrived refugee and immigrant children compared with their Ontario-born peers.

**Design, Setting, and Participants:**

This population-based cohort study took place in Ontario, Canada, and included immigrant children who arrived between 2008 and 2017 and Ontario-born children. Participants were aged 0 to 14 years and followed up for 4 years after index date (ie, 1 year after health care eligibility). Data were analyzed between November 2023 and December 2025.

**Exposure:**

Government-assisted refugees (GARs), privately sponsored refugees (PSRs), successful asylum-seekers (referred to as protected persons [PPs] in Canada), nonrefugee immigrant (NRIs), each matched 1:4 to Ontario-born children by age, sex, and urban area.

**Main Outcomes and Measures:**

The percentage of minor illness ED visits (ie, primary care plus ED visits for equivalent reasons) in the first and second 2-year periods after eligibility was calculated for each child with at least 1 minor illness visit. The difference in mean percentage was modeled using linear regression and compared each immigrant group to Ontario-born children adjusting for morbidity, material resources quintile, and primary care affiliation.

**Results:**

Overall, 458 597 children were included (mean [SD] age, 8.0 [4.3] years; 221 237 females [48%]; 237 360 males [52%]; GARs, 10 211 [2.23%]; PSRs, 7810 [1.70%]; PPs, 11 540 [2.52%]; NRIs, 83 537 [18.22%]; Ontario-born matches, 345 499 [75.34%]). In the first 2 years, immigrant groups had significantly lower adjusted differences in the mean percentage of minor illnesses seen in the ED compared with their Ontario-born matches (GARs: −5.11% [95% CI, −5.63% to −4.57%]; PSRs: −5.24% [95% CI, −5.80% to −4.67%]; PPs: −3.37% [95% CI, −3.86% to −2.87%]; NRIs: −4.24% [95% CI, −4.40% to −4.09%]). In the second 2 years, differences were attenuated but remained significantly lower.

**Conclusions and Relevance:**

In this cohort study, refugee and nonrefugee immigrant children in their early resettlement period were less likely to use the ED than Ontario-born children for minor illnesses, suggesting adequate access to primary care.

## Key Points

**Question:**

Do recent immigrant children have a higher mean percentage of all minor illness visits seen in emergency departments (EDs) than Ontario-born children?

**Findings:**

In this population-based matched cohort study including 458 597 children, adjusted differences in the mean percentage of all minor illnesses seen in the ED were significantly lower for refugee groups (government-assisted refugees,−5.11%; privately sponsored refugees, −5.24%; successful asylum-seekers, −3.73%) and nonrefugee immigrants (−4.24%) compared with Ontario-born children.

**Meaning:**

These findings suggest that recently arrived immigrant children were not more likely to use emergency departments over primary care for minor illness compared with Ontario-born children.

## Teaser

This cohort study examines refugee and immigrant access to primary care by analyzing the percentage of all minor illness visits seen in emergency departments (EDs) in Ontario, Canada.

## Introduction

The use of emergency departments (EDs) for minor illnesses is an important indicator measuring inadequate primary care access, potential issues with care management and patient decision-making.^1^ Both the US^2^ and Canada^3,4^ are in the midst of a physician shortage with decreased primary care access disproportionately affecting disadvantaged populations. Recent immigrants and refugees of all ages may experience additional challenges in accessing primary care including unfamiliarity with the local health care system, competing priorities, as well as geographic and sociocultural differences (eg, language discordance), all of which may lead to use of EDs for minor illnesses.^5,6,7^ Given Canada currently admits large numbers of permanent residents (approximately 400 000 individuals per year in recent years) who are also eligible for publicly funded health insurance, it is an ideal location to study health care navigation among recent immigrants.

In Canada, there are multiple pathways to permanent residency for immigrants and refugees (Table 1). Resettled refugee streams include government-assisted refugees (GARs), blended-visa office referred refugees (BVORs) and privately sponsored refugees (PSRs). Health care navigation support for GARs is provided by settlement workers in the first year^8^ and often within community health centers (CHCs) in Ontario, where primary care and settlement services are often co-located. Health care navigation support for PSRs or BVORs depends on the knowledge and resources of the private sponsorship group,^9^ who described access to health care as the second biggest challenge (after housing) in the early resettlement period.^10^ The private sponsorship program, pioneered in Canada, is receiving increased international attention^11^ including the launch of a similar program in the US^12^ (prior to being suspended in 2025) and other countries.^13^ Successful asylum-seekers (known as protected persons [PPs] in Canada) are offered little to no formal health care navigation support. Nonrefugee immigrants (approximately 90% of Canada’s intake of permanent residents) are economic immigrants, where adults are selected based on the ability to speak Canadian official languages, high educational attainment, and lower levels of anticipated health care need, or are sponsored by family in Canada.^14^

**Table 1.** Canadian Immigration Pathways, Selection Criteria, and Early Health Care Navigation Supports

Characteristic	Resettled refugee			Asylum-seeker, protected persons	Nonrefugee immigrants	
	Government-assisted refugees	Blended visa office referred refugees	Privately sponsored refugees		Sponsored family	Economic immigrants
Approximate % of national annual intake of permanent residents	5	5	5	5	30	60
Referring agency	UNHCR or migration agency	UNHCR or migration agency	Private sponsors, UNHCR or migration agency, matched to sponsors	Self-selected	Not applicable	Not applicable
Health selection criteria applied	No	No	No	No	For some groups^a^	Yes^a^
Other selection criteria applied	Yes^b^	Yes^b^	Yes^b^	Yes	Yes	Yes^c^
Health care navigation in first year	Settlement worker, community health centers	Sponsorship group	Sponsorship group	Little to no formal navigation offered	No formal navigation offered	No formal navigation offered
Financial support in the first year	Yes, government support	Yes, split between government and sponsor	Yes, sponsoring group	None	None	None

Abbreviation: UNHCR, United Nations High Commissioner for Refugees.

^a^ Can be rejected for immigration on the basis of preexisting health conditions estimated to lead to high levels of health care use.

^b^ UNHCR resettlement criteria can include aspects such as medical need, surviving violence and trauma, women at risk and heightened vulnerability, and urgency of resettlement need.

^c^ A points-based selection system is applied to economic immigrants based on the ability to speak Canadian official languages, high educational attainment and Canadian work experience.

While support for the healthy immigrant effect among immigrant children in Canada is mixed,^15^ all children are susceptible to minor illnesses (eg, respiratory infections, injuries) which may require access to health care services. Our study had 2 aims. First, we examined differences in the mean percentage of all minor illnesses seen in the ED in the first 4 years among recently arrived refugees and nonrefugee immigrant children and youth compared with those born in Ontario. We hypothesized that all immigrant groups would have a higher mean percentage of all minor illnesses seen in the ED compared with Ontario-born children, associated with challenges in navigating primary care in the early years of resettlement with relative differences decreasing with time. We also hypothesized variability among the groups given differences in underlying health needs and health care navigation support. In our second aim, we examined whether primary care affiliation explained differences between groups.

## Methods

### Study Design and Population

We conducted a retrospective, population-based, matched cohort study linking multiple datasets, including the provincial health insurance registry, the federal immigration permanent resident file, and health care service data (eTable 1 in Supplement 1). These datasets were linked using unique encoded identifiers and analyzed at ICES. ICES is an independent, nonprofit research institute whose legal status under Ontario’s health information privacy law allows it to collect and analyze health care and demographic data, without consent, for health system evaluation and improvement. The use of data in this project is authorized under section 45 of Ontario’s Personal Health Information Protection Act and does not require review by a research ethics board. We followed the Strengthening the Reporting of Observational Studies in Epidemiology (STROBE) reporting guideline and REporting of Studies Conducted Using Observational Routinely-Collected Health Data (RECORD) Statement.^16,17^

This study included all refugee and nonrefugee immigrant children aged 0 to 14 years, who received permanent residency status in Canada and resided in urban Ontario between April 1, 2008, and September 30, 2017. The health care eligibility date closest to date of permanent residency was chosen, and for those arriving at the end of arrival period, we allowed for an additional 6 months to become eligible for health care (until March 31, 2018). The observation window for outcomes started 1 year after the health care eligibility date (index date) to allow for a more stable profile of health care use and primary care enrollment and ended 4 years after index with a maximum follow-up date of March 31, 2023. Ontario’s health care system is publicly funded with revenue raised through taxation and covers most medically necessary services. eTable 2 in Supplement 1 contains details about all study variables.

### Exposure

The primary exposure was immigration pathway, specifically those arriving as GARs, PSRs or BVORs, PPs, and NRIs. Each immigrant group was separately matched with a ratio of 1:4 on Census Metropolitan Area (major urban area), sex and age at index date, to children born in Ontario whose birthing parent was not in the immigration database.

### Outcomes

The primary outcome was conceptualized as a measure of ED visits more appropriately seen in primary care, and therefore potentially preventable. This was operationalized as a percentage calculated for each child with the numerator being the number of all minor ED visits, defined as level 4 and 5 (less urgent and nonurgent) on the Canadian Triage and Acuity Scale (CTAS)^18^ and with home discharge (conceptually equivalent to primary care sick visits); while the denominator was all minor illness visits, both primary care sick visits (excluding codes in eTables 3 and 4) and minor ED visits. In sensitivity analyses, we restricted the numerator to minor ED visits with a family practice sensitive condition (eg, otitis media) (eTable 5 in Supplement 1)^19^ thought to be best managed in a primary care office.

To ensure that we examined outcomes that could reflect poor access to primary care among those with at least 1 minor illness, we also examined complementary outcomes: (1) the mean number of both high acuity ED visits which could be the result of deferred care, defined as levels 1, 2, and 3 (resuscitation, emergent and urgent respectively), (2) the mean number of routine primary care visits, and (3) the proportion with a routine primary care visit (eTables 3 and 4 in Supplement 1). To further ensure that our decision to have a cohort with at least 1 minor illness visit did not bias our sample, we also analyzed the potential for delayed care and access to routine care among those immigrant children with no minor illnesses. All outcomes were ascertained in 2-year intervals. After the first 2 years, those who died were censored to obtain a revised denominator for the final 2 years.

### Covariates

Covariates included sociodemographic and immigration related characteristics. Material resources quintile,^20^ considered a measure of neighborhood socioeconomic status, was based on the residential neighborhood (dissemination area). Age, sex, and material resources quintile were reported at index and 2 years after. Prevalence of at least 1 major comorbidity was defined using the Johns Hopkins Adjusted Clinical Group System (version 10) Aggregated Diagnosis Groups (ADGs). Morbidity and primary care affiliation were measured using data on health care use and patient rostering, respectively, collected in the year prior to the first and second index dates. Region of birth, knowledge of official languages, age at arrival and arrival year were reported as of the date of permanent residency.

### Statistical Analyses

We examined whether the frequencies of baseline characteristics were balanced between the immigration and Ontario-born groups (comparison population) using SD greater than 0.1.^21^ To identify the analytic population for our primary analyses, we first identified children with at least 1 minor illness visit to primary care or the ED. Then to maintain the integrity of the each matched group (1) among immigrant children, we excluded immigrants for whom all their Ontario-born matches had no minor illnesses, and (2) among Ontario-born children, we excluded all 4 Ontario-born matches if their matched immigrant child had no minor illnesses. These exclusions resulted in some matched groups having matching ratios of less than 4:1.

Linear regression with generalized estimating equations, assuming a normal distribution and identity link function, was used to estimate the difference in the mean percentage of all minor illnesses seen in the ED between each immigrant group and Ontario-born matched comparison, with matched groups as the clustering variable. Separate models were estimated for each immigrant group and Ontario-born matched comparator, and for the first and the second 2 years of the follow-up period. Models were first adjusted for any major morbidity and material resources quintile. We additionally adjusted for primary care affiliation to assess if differences between exposure groups were explained by this variable. Models were replicated for sensitivity analyses, focusing on minor illnesses seen in the ED with a family practice sensitive condition. Data were analyzed between November 2023 and December 2025. Statistical significance was set at *P* < .05, and all tests were 2-sided. Data were examined using SAS Enterprise Guide 8.3 (2024/2025) (SAS Institute). Statistical analyses of complementary measures and secondary outcomes were not conducted.

## Results

After exclusions (eFigure 1 in Supplement 1), 737 695 children (mean [SD] age, 8.3 [4.2] years; 353 875 [48%] female) were eliglible. eTable 6 in Supplement 1 further describes the cohort characteristics before restricting to population with at least 1 minor illness to the ED or primary care. At index, 458 497 children (mean [SD] age, 8.0 [4.3] years; 221 237 females [48%]) were included. There were 10 211 GARs (2.23%); 7810 PSRs and BVORs (1.70%); 11 540 PPs (2.52%); 83 537 NRIs (18.22%), and 345 499 Ontario-born matched children (75.34%) with at least 1 minor illness seen in primary care or the ED (Table 1). eTable 7 in Supplement 1 summarizes cohort characteristics at 2 years after index. For the primary analyses of children with at least 1 minor illness visit, distribution of age categories and sex remained balanced between each immigrant category and their Ontario-born matches.

At index, immigrant groups were 3 to 4 times more likely to reside in neighborhoods in the lowest material resources quintile compared with their Ontario-born matches, with proportions similar or dropping slightly in the second 2 years (eTable 7 in Supplement 1). There were no differences in the proportion with a major morbidity measured at index or 2 years after index between each immigrant group and their matched group. GARs were much more likely to have visited a community health center than their Ontario-born matches (2105 of 10 211 [20.6%] vs 275 of 31 017 [0.9%]), while PSRs or BVORs (610 of 7810 [7.8%] vs 201 of 23 467 [0.9%]), PPs (700 of 11 540 [6.1%] vs 162 of 34 778 [0.5%]), and NRIs (1853 of 83 537 [2.2%] vs 1466 of 256 237 [0.6%]) were slightly more likely than their Ontario-born matches to have visited a community health center. All refugee groups were less likely to be enrolled in a comprehensive model or have a pediatrician as a primary care physician compared with their matches (Table 2). In the subsequent 2 years, the proportion enrolled in a comprehensive model increased for all immigrant groups while the proportion visiting other primary care physicians decreased.

**Table 2.** Characteristics at Index of Immigrant Children and Youth Arriving in Ontario Between 2008 and 2017 and Their Ontario-Born Matches With at Least 1 Minor Illness in the First 2 Years Since the Index Date

Characteristics	Participants, No. (%)		StD^a^	Participants, No. (%)		StD^a^	Participants, No. (%)		StD^a^	Participants, No. (%)		StD^a^
	GARs	Ontario-Born matches		PSRs or BVORs	Ontario-born matches		PPs	Ontario-born matches		NRIs	Ontario-born matches	
Total	10 211	31 017	NA	7810	23 467	NA	11 540	34 778	NA	83 537	256 237	NA
Age at index, y												
<1	0	0	0	0	0	0	0	0	0	9 (0)	29 (0)	0
1-3	1835 (18.0)	5983 (19.3)	0.03	1394 (17.8)	4448 (19.0)	0.03	744 (6.4)	2377 (6.8)	0.02	16 696 (20.0)	53 741 (21.0)	0.02
4-6	2335 (22.9)	7164 (23.1)	0.01	1803 (23.1)	5409 (23.0)	0	2657 (23.0)	8172 (23.5)	0.01	18 706 (22.4)	57 635 (22.5)	0
7-9	2270 (22.2)	6512 (21.0)	0.03	1695 (21.7)	4856 (20.7)	0.02	2866 (24.8)	8346 (24.0)	0.02	16 160 (19.3)	47 840 (18.7)	0.02
10-12	1984 (19.4)	5858 (18.9)	0.01	1470 (18.8)	4352 (18.5)	0.01	2831 (24.5)	8393 (24.1)	0.01	15 389 (18.4)	45 709 (17.8)	0.02
13-15	1781-1784^b^	5491 (17.7)	0.01	1406 (18.0)	4269 (18.2)	0	2317 (20.1)	7086 (20.4)	0.01	15 038 (18.0)	46 354 (18.1)	0
≥16	1-5^c^	9 (0)	0	42 (0.5)	133 (0.6)	0	125 (1.1)	404 (1.2)	0.01	1539 (1.8)	4929 (1.9)	0.01
Sex												
Female	4984 (48.8)	15 299 (49.3)	0.01	3791 (48.5)	11 501 (49.0)	0.01	5668 (49.1)	17 330 (49.8)	0.01	39 764 (47.6)	122 900 (48.0)	0.01
Male	5227 (51.2)	15 718 (50.7)	0.01	4019 (51.5)	11 966 (51.0)	0.01	5872 (50.9)	17 448 (50.2)	0.01	43 773 (52.4)	133 337 (52.0)	0.01
Material Resources Quintile at index												
1 (most)	218 (2.1)	9731 (31.4)	0.85^a^	405-40^b^	6458 (27.5)	0.63^a^	452 (3.9)	8848 (25.4)	0.64^a^	7639 (9.1)	60 171 (23.5)	0.40^a^
2	343 (3.4)	7020 (22.6)	0.60^a^	658 (8.4)	5668 (24.2)	0.44^a^	601 (5.2)	8408 (24.2)	0.56^a^	10 163 (12.2)	61 687 (24.1)	0.31^a^
3	1241 (12.2)	4801 (15.5)	0.10^a^	949 (12.2)	4257 (18.1)	0.17^a^	985 (8.5)	6711 (19.3)	0.31^a^	13 275 (15.9)	51 751 (20.2)	0.11^a^
4	1991 (19.5)	4342 (14.0)	0.15^a^	1580 (20.2)	3500 (14.9)	0.14^a^	2147 (18.6)	5526 (15.9)	0.07	19 632 (23.5)	43 431 (16.9)	0.16^a^
5 (least)	6418 (62.9)	5117 (16.5)	1.08^a^	4212 (53.9)	3571 (15.2)	0.89^a^	7349 (63.7)	5264 (15.1)	1.14^a^	32 803 (39.3)	39 053 (15.2)	0.56^a^
Missing	0	6 (0)	0.02	1-5^c^	13 (0.1)	0.01	6 (0.1)	21 (0.1)	0	25 (0)	144 (0.1)	0.01
Major morbidity at index												
No	9109 (89.2)	28 360 (91.4)	0.08	7172 (91.8)	21 452 (91.4)	0.02	10 678 (92.5)	31 892 (91.7)	0.03	77 717 (93.0)	234 193 (91.4)	0.06
Yes	1102 (10.8)	2657 (8.6)	0.08	638 (8.2)	2015 (8.6)	0.02	862 (7.5)	2886 (8.3)	0.03	5820 (7.0)	22 044 (8.6)	0.06
Primary care affiliation at index												
CHC	2105 (20.6)	275 (0.9)	0.67^a^	610 (7.8)	201 (0.9)	0.35^a^	700 (6.1)	162 (0.5)	0.32^a^	1853 (2.2)	1466 (0.6)	0.14^a^
Comprehensive care	4017 (39.3)	22 237 (71.7)	0.69^a^	3232 (41.4)	16 105 (68.6)	0.57^a^	4851 (42.0)	23 654 (68.0)	0.54^a^	42 438 (50.8)	169 553 (66.2)	0.32^a^
Pediatrician	216 (2.1)	1460 (4.7)	0.14^a^	141 (1.8)	1510 (6.4)	0.23^a^	260 (2.3)	2460 (7.1)	0.23^a^	1407 (1.7)	20 415 (8.0)	0.30^a^
Other PCP	3331 (32.6)	4559 (14.7)	0.43^a^	3046 (39.0)	3622 (15.4)	0.55^a^	4216 (36.5)	5225 (15.0)	0.51^a^	25 839 (30.9)	41 927 (16.4)	0.35^a^
No PCP	542 (5.3)	2486 (8.0)	0.11^a^	781 (10.0)	2029 (8.6)	0.05	1513 (13.1)	3277 (9.4)	0.12^a^	12 000 (14.4)	22 876 (8.9)	0.17^a^
**Immigration-related characteristics**												
Age at arrival, y												
<1	511 (5.0)	NA	NA	335 (4.3)	NA	NA	33 (0.3)	NA	NA	4129 (4.9)	NA	NA
1-3	2103 (20.6)	NA	NA	1724 (22.1)	NA	NA	1002 (8.7)	NA	NA	21 220 (25.4)	NA	NA
4-6	2343 (22.9)	NA	NA	1777 (22.8)	NA	NA	2883 (25.0)	NA	NA	17 399 (20.8)	NA	NA
7-9	2193 (21.5)	NA	NA	1589 (20.3)	NA	NA	2918 (25.3)	NA	NA	15 615 (18.7)	NA	NA
10-12	1902 (18.6)	NA	NA	1478 (18.9)	NA	NA	2849 (24.7)	NA	NA	15 189 (18.2)	NA	NA
13-15	1159 (11.4)	NA	NA	907 (11.6)	NA	NA	1855 (16.1)	NA	NA	9985 (12.0)	NA	NA
Arrival year												
2008-2010	1967 (19.3)	NA	NA	1453 (18.6)	NA	NA	3448 (29.9)	NA	NA	32 076 (38.4)	NA	NA
2011-2014	2429 (23.8)	NA	NA	1733 (22.2)	NA	NA	5000 (43.3)	NA	NA	33 384 (40.0)	NA	NA
2015-2017	5815 (56.9)	NA	NA	4624 (59.2)	NA	NA	3092 (26.8)	NA	NA	18 077 (21.6)	NA	NA
Birth region												
Central Africa	252 (2.5)	NA	NA	25 (0.3)	NA	NA	256 (2.2)	NA	NA	300 (0.4)	NA	NA
Western Africa	62 (0.6)	NA	NA	23 (0.3)	NA	NA	733 (6.4)	NA	NA	1940 (2.3)	NA	NA
East Africa	1084 (10.6)	NA	NA	655 (8.4)	NA	NA	1085 (9.4)	NA	NA	1011 (1.2)	NA	NA
Southern Africa	74 (0.7)	NA	NA	50 (0.6)	NA	NA	63 (0.5)	NA	NA	322 (0.4)	NA	NA
Middle East	7104 (69.6)	NA	NA	5370 (68.8)	NA	NA	974 (8.4)	NA	NA	12 847 (15.4)	NA	NA
North Africa	194 (1.9)	NA	NA	258 (3.3)	NA	NA	210 (1.8)	NA	NA	3041 (3.6)	NA	NA
Central America	0 (0.0)	NA	NA	1-5^b^	NA	NA	407 (3.5)	NA	NA	842 (1.0)	NA	NA
South America	106 (1.0)	NA	NA	45 (0.6)	NA	NA	846 (7.3)	NA	NA	2033 (2.4)	NA	NA
Caribbean	1-5^b^	NA	NA	1-5^b^	NA	NA	859 (7.4)	NA	NA	3782 (4.5)	NA	NA
North America	1-5^b^	NA	NA	17 (0.2)	NA	NA	1594 (13.8)	NA	NA	4477 (5.4)	NA	NA
East Asia	1-5^b^	NA	NA	1-5^b^	NA	NA	968 (8.4)	NA	NA	7017 (8.4)	NA	NA
Australasia/Oceania and Asia unspecified	0 (0.0)	NA	NA		NA	NA	1-5^b^	NA	NA	375 (0.4)	NA	NA
Southeast Asia	314 (3.1)	NA	NA	101 (1.3)	NA	NA	35-39^c^	NA	NA	13 402 (16.0)	NA	NA
South Asia	670 (6.6)	NA	NA	968 (12.4)	NA	NA	2597 (22.5)	NA	NA	25 087 (30.0)	NA	NA
Eastern Europe	128 (1.3)	NA	NA	28 (0.4)	NA	NA	563 (4.9)	NA	NA	3234 (3.9)	NA	NA
Europe other	213 (2.1)	NA	NA	258 (3.3)	NA	NA	345 (3.0)	NA	NA	3827 (4.6)	NA	NA
Official language												
English	1012 (9.9)	NA	NA	1559 (20.0)	NA	NA	6445 (55.8)	NA	NA	38 097 (45.6)	NA	NA
French	88 (0.9)	NA	NA	39 (0.5)	NA	NA	657 (5.7)	NA	NA	891 (1.1)	NA	NA
Bilingual	33 (0.3)	NA	NA	25 (0.3)	NA	NA	356 (3.1)	NA	NA	887 (1.1)	NA	NA
Neither	8801 (86.2)	NA	NA	6056 (77.5)	NA	NA	3956 (34.3)	NA	NA	43 067 (51.6)	NA	NA
Missing	277 (2.7)	NA	NA	131 (1.7)	NA	NA	126 (1.1)	NA	NA	595 (0.7)	NA	NA
Health care eligibility prior to arrival date (possible history of being a temporary resident)^d^												
Yes	218 (2.1)	NA	NA	90 (1.2)	NA	NA	5295 (45.9)	NA	NA	3910 (4.7)	NA	NA
No	9993 (97.9)	NA	NA	7720 (98.8)	NA	NA	6245 (54.1)	NA	NA	79 627 (95.3)	NA	NA

Abbreviation: BVOR, blended-visa office referred refugees; CHC, community health centers; GARs, government-assisted refugees; NA, not applicable; NRI, nonrefugee immigrant; PP, protected persons; PSR, privately sponsored refugees; NA, not applicable; StD, standardized difference.

^a^ StD < 0.1 indicates variable is balanced between immigrant group and matched Ontario-born population. The StD is estimated only for variables available for both immigrants and the Ontario-born population.

^b^ Small cells (ie, <6) are suppressed in accordance with ICES policy.

^c^ Nonmissing data reported as ranges without percentage to reduce risk of reidentification in accordance with ICES policy.

^d^ If both health care eligibility and follow-up time for outcomes starts before the arrival date for permanent residency, the period of health care use prior to permanent residency and while a temporary resident is captured in this study.

**Figure 1.**
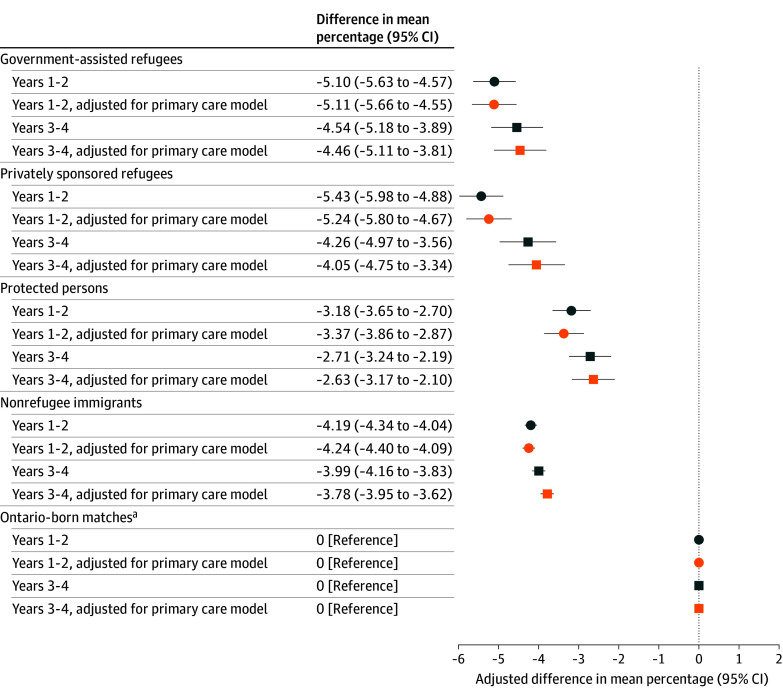
Dot Plot Showing Differences in the Mean Percentage of All Minor Illnesses in the Emergency Department

After adjustment for major morbidity and material resources quintile (Figure 1 and eTable 8 in Supplement 1), GARs and PSR or BVORs had significantly lower mean (95% CI) percentages of minor illnesses seen in the ED compared with their Ontario-born matches (−5.10% [95% CI, −5.63% to −4.57%] and −5.43% [95% CI, −5.98% to −4.88%], respectively) followed by NRIs (−4.19% [95% CI, −4.34% to −4.04%]) and PPs (−3.18% [95% CI, −3.65% to −2.70%]). In the second 2 years after index, findings were slightly attenuated but were not significantly different than the first 2 years. Adjustment for primary care affiliation did not substantially attenuate differences.

**Figure 2.**
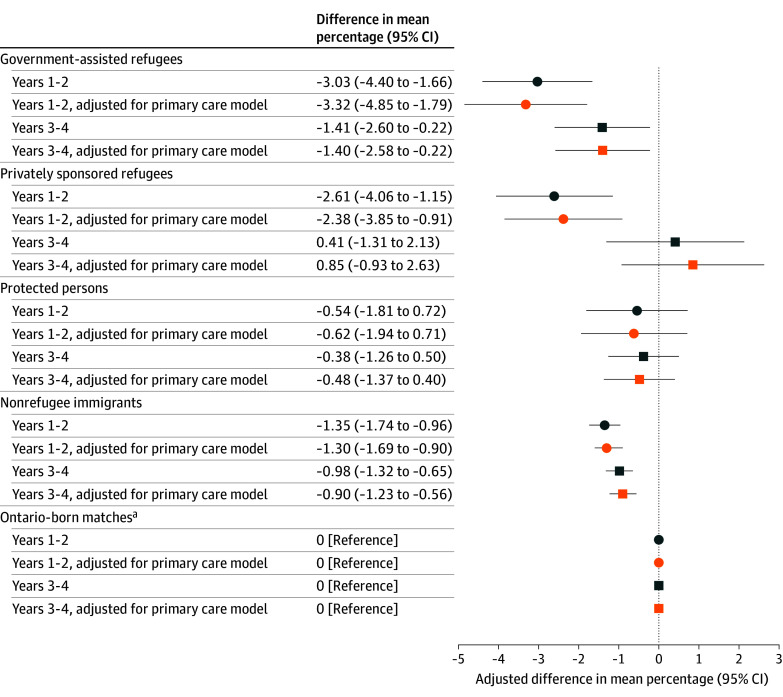
Dot Plot Showing Differences in the Mean Percentage of All Minor Illnesses in the Emergency Department With a Family Practice Sensitive Condition

Regarding minor illness visits to the ED with a family practice sensitive condition, for the first 2 years, models demonstrated lower differences in the mean (95% CI) percentage for most immigrant groups (GAR, −3.03 [95% CI, −4.40 to −1.66]; PSR or BVOR, −2.61 [95% CI, −4.06 to −1.15]; NRIs, −1.35 [95% CI, −1.74 to −0.96]), but with no difference for PPs compared with Ontario-born matches (Figure 2 and eTable 9 in Supplement 1). For the second 2 years after index, differences were not significantly different than the first 2 years but were attenuated for PSR or BVORs while NRIs continued to have lower proportions. Adjustment for primary care affiliation did not attenuate findings in the first or second 2 years.

Among those with at least 1 minor illness visits all immigrant groups had a higher mean number of routine primary care visits compared with their Ontario-born matches (eTable 10 in Supplement 1). In the second 2 years, this mean decreased for all groups but remained higher among immigrant groups. The mean number of acute ED visits in the first 2 years was slightly lower for immigrant groups than their matches. These values were similar in the second 2 years.

For secondary outcomes (eTable 11 in Supplement 1) among children with no minor illness visits to primary care or the emergency department in the first or second 2 years, there were no differences in the mean number of acute ED visits between refugee groups and their matches. The mean number of routine primary care visits in the first 2 years was slightly higher among GARs and PSR or BVORs compared with their Ontario-born matches. Both secondary outcome means were lower among NRIs. In the second 2 years, the mean number of routine primary care visits dropped for all groups (compared with the first 2 years) and PPs and NRIs had a lower mean number of routine primary care visits compared with their Ontario-born matches.

## Discussion

In the first 2 years of follow-up, we found that refugee and immigrant children with at least 1 minor illness visit had significantly lower mean percentages of all minor illnesses seen in the emergency department (ED) compared with their Ontario-born peers. Those with health care navigation support during early settlement (GARs, PSRs or BVORs) had larger negative differences in the mean percentage of all minor illnesses seen in the ED, than those with no navigation support (PPs, NRIs). Matched Ontario-born children (with mostly nonimmigrant mothers) whom we assumed could leverage their knowledge and familiarity with the health care system to better facilitate primary care navigation all had significantly higher mean percentages of all minor illnesses seen in the ED than their immigrant counterparts. Slightly attenuated differences in the mean percentage in the second 2 years suggesting the use of the ED for minor illnesses increased for immigrants by shifting closer to that of their Ontario-born matches. Primary care affiliation did not explain differences in the outcome between immigrant and Ontario-born children. When we examined the mean percentage of minor ED visits with a family practice sensitive condition in the first 2 years, the difference in the mean percentage remained significantly low for GARs and PSRs or BVORs while PPs were nonsignificant suggesting PP’s experiences of health care navigation and settlement are different than that of resettled refugees. In the second 2 years for GARs and PSRs or BVORs, differences approached and become nonsignificant respectively, suggesting change occurring over time that was independent of changes in primary care affiliation. Among those with no minor illness visits to primary care or the emergency department, delayed care among immigrant groups was not evident while access to routine care was higher among resettled refugee groups and lower among NRIs. Our findings were largely contrary to our hypotheses.

Contrary to our study, a 2024 systematic review^22^ examining ED use in migrant and nonmigrant populations, identified 4 studies from Switzerland, Germany, Italy, and Singapore^7,23,24,25^ where migrants tended to visit the ED for less urgent conditions more often than host populations. When comparing general ED use,^22^ studies from the US,^26^ Spain,^27,28^ Italy,^7^ and Switzerland^29^ were inconsistent, with only the latter finding lower ED use among recent asylum-seeking pediatric patients. A 2018 systematic review^6^ of health care use in migrant children and mixed-aged populations, also cited heterogenous evidence; with findings similar to ours among refugees in Calgary, Canada^30^ and among migrants in Norway,^31^ and contradictory findings among infants born to immigrant mothers in Italy.^32^

To our knowledge, operationalization of our primary outcome appears unique to the literature, making direct comparison challenging. We could identify only 1 study which also examined ED use among children in the early resettlement period.^29^ Heterogeneity in the definition and measurement of minor ED visits can lead to inappropriate interpretations of study results.^33^ If the definition of a minor ED visit is solely based on triage-score, some nonurgent ED visits are appropriate. Indeed, when we examined the top 5 diagnoses in the ED using our primary outcome, some conditions were those where the ED may be the best venue (ie, lacerations) (eTable 12 in Supplement 1). We addressed the question of validity of our primary outcome by conducting a sensitivity analysis in which the outcome was limited to family practice sensitive conditions. These top 5 diagnoses were all unspecified infections which are usually appropriately seen by primary care (eTable 13 in Supplement 1). These results provide support that the family practice sensitive condition definition may be a more appropriate representation of preventable ED visits than the triage score alone.

In our family practice sensitive condition analyses, for GARs and PSRs or BVORs in the second 2-year period, we found a pronounced increase in preventable ED visits, compared with the first 2 years. This difference may be associated with a reduction of resettlement financial support and the known challenges of resettled refugees finding suitable employment,^34^ which may hinder seeking primary care during office hours. Diminished health care navigation support from settlement workers and sponsors could also explain this increased use of ED for minor issues in the second 2 years.

In high-income countries it is commonly perceived that migrants, particularly refugees and asylum-seekers, are undeserving of publicly funded services, with sentiments often invoked to justify xenophobic policies^35,36,37,38,39^ and decrease support for migration. The findings of our study add to a growing body of evidence disproving extensive ED usage among migrants.^20,30,31,40^ Research from Greece, Sweden, and Germany demonstrated that inclusive health care for migrants not only improved health but also generated cost savings.^41^

### Limitations

The use of administrative data and the retrospective design limit our ability to measure potentially important variables, including individual household income, parent or guardian education, or the specific reasons for caregivers’ decision to present to the ED with a minor illness. The generalizability of our findings to undocumented migrants or asylum seekers awaiting their refugee hearing and to countries with less comprehensive permanent residency programs and less inclusive health systems is uncertain. Additionally, findings may differ in contexts with differently funded health care systems.

## Conclusions

The findings of this population-based study are surprising. Despite deepening constraints on the primary care system, recently arrived immigrant children had lower mean percentages of all minor illness visits seen in the ED compared with Ontario-born children. The variation in differences observed across comparisons in the first 2 years may be explained by differences in receipt of health care navigation support. In the second 2 years after arrival, attenuated differences for all comparisons suggested other factors, including possible shifts in financial support and employment patterns for some refugee groups, may be influencing health care–seeking behavior for minor illnesses more toward the ED and away from primary care.

## Notes

Supplement 1. **eFigure.** Study flow diagram **eTable 1.** Description of linked administrative databases **eTable 2.** List of variables and their definitions **eTable 3.** OHIP core primary care service fee codes and routine primary care visit diagnosis codes **eTable 4.** Community health centre (CHC) routine primary care service visit types and related diagnosis codes **eTable 5.** List of *ICD-10* CA diagnosis codes indicating family practice sensitive conditions **eTable 6.** Characteristics of immigrant children and youth compared with their Ontario-born matches **eTable 7.** Characteristics 2 years after index of immigrant children and youth arriving in Ontario 2008-2017 and their Ontario-born matches with at least one minor illness visit to primary care or the emergency department in the second 2 years after the index **eTable 8.** Modelling the difference in the mean percentage of all minor illness visits seen in the ED (among those who have at least 1 minor illness) and 95% CI for all linear regression model coefficients, comparing immigrant groups to their Ontario-born matched group **eTable 9.** Modelling the difference in the mean percentage of all minor illness visits seen in the ED with a Family Practice Sensitive Condition (among those who have at least one minor illness) and 95% CI for all linear regression model coefficients, comparing immigrant groups to their Ontario-born matched group **eTable 10.** Primary outcome, sensitivity outcome and complementary measures among immigrant children and youth compared to their Ontario-born matches, with at least one minor illness seen in the emergency department or primary care, in Years 1 and 2 and Years 3 and 4 **eTable 11.** Secondary outcomes among immigrant children and youth compared to their Ontario-born matches, with no minor illnesses (in either the emergency department or primary care), Years 1 and 2 and Years 3 and 4 **eTable 12.** Frequencies for the top 5 *ICD-10* chapters for most responsible diagnosis for ED triage 4 to 5 visits for each exposure group and their matched group **eTable 13.** Frequencies for the top 5 *ICD-10* chapters for most responsible diagnosis for ED triage 4 to 5 visits with a family practice sensitive condition for each exposure group and their matched group
eReferences.

Supplement 2.
Data Sharing Statement
