# Supplementary material for: Emergency Department Visits for Minor Illnesses Among Recent Refugee and Immigrant Children
Source: JAMA Netw Open. 2026 Feb 27;9(2):e2560070. doi: 10.1001/jamanetworkopen.2025.60070 (PMC12949439; doi:10.1001/jamanetworkopen.2025.60070)
Supplement: Supplement 1. — eFigure. Study flow diagram eTable 1. Description of linked administrative databases eTable 2. List of variables and their definitions eTable 3. OHIP core primary care service fee codes and routine primary care visit diagnosis codes eTable 4. Community health centre (CHC) routine primary care service visit types and related diagnosis codes eTable 5. List of ICD-10 CA diagnosis codes indicating family practice sensitive conditions eTable 6. Characteristics of immigrant children and youth compared with their Ontario-born matches eTable 7. Characteristics 2 years after index of immigrant children and youth arriving in Ontario 2008-2017 and their Ontario-born matches with at least one minor illness visit to primary care or the emergency department in the second 2 years after the index eTable 8. Modelling the difference in the mean percentage of all minor illness visits seen in the ED (among those who have at least 1 minor illness) and 95% CI for all linear regression model coefficients, comparing immigrant groups to their Ontario-born matched group eTable 9. Modelling the difference in the mean percentage of all minor illness visits seen in the ED with a Family Practice Sensitive Condition (among those who have at least one minor illness) and 95% CI for all linear regression model coefficients, comparing immigrant groups to their Ontario-born matched group eTable 10. Primary outcome, sensitivity outcome and complementary measures among immigrant children and youth compared to their Ontario-born matches, with at least one minor illness seen in the emergency department or primary care, in Years 1 and 2 and Years 3 and 4 eTable 11. Secondary outcomes among immigrant children and youth compared to their Ontario-born matches, with no minor illnesses (in either the emergency department or primary care), Years 1 and 2 and Years 3 and 4 eTable 12. Frequencies for the top 5 ICD-10 chapters for most responsible diagnosis for ED triage 4 to 5 visits for each exposure group and the [file jamanetwopen-e2560070-s001.pdf]

Supplemental Online Content

Wanigaratne S, Brandenberger J, Lu H, et al. Emergency department visits for minor illnesses among recent refugee and immigrant children. *JAMA Netw Open*. 2026;9(2):e2560070. doi:10.1001/jamanetworkopen.2025.60070

eFigure. Study flow diagram

eTable 1. Description of linked administrative databases

eTable 2. List of variables and their definitions

eTable 3. OHIP core primary care service fee codes and routine primary care visit diagnosis codes

eTable 4. Community Health Centre (CHC) routine primary care service visit types and related diagnosis codes

eTable 5. List of *ICD-10 CA* diagnosis codes indicating Family Practice Sensitive Conditions

eTable 6. Characteristics of immigrant children and youth compared with their Ontario-born matches

eTable 7. Characteristics 2 years after index of immigrant children and youth arriving in Ontario 2008-2017 and their Ontario-born matches with at least one minor illness visit to primary care or the emergency department in the second 2 years after the index

eTable 8. Modelling the difference in the mean percentage of all minor illness visits seen in the ED (among those who have at least 1 minor illness) and 95% CI for all linear regression model coefficients, comparing immigrant groups to their Ontario-born matched group

eTable 9. Modelling the difference in the mean percentage of all minor illness visits seen in the ED with a Family Practice Sensitive Condition (among those who have at least one minor illness) and 95% CI for all linear regression model coefficients, comparing immigrant groups to their Ontario-born matched group

eTable 10. Primary outcome, sensitivity outcome and complementary measures among immigrant children and youth compared to their Ontario-born matches, with at least one minor illness seen in the emergency department or primary care, in Years 1 and 2 and Years 3 and 4

eTable 11. Secondary outcomes among immigrant children and youth compared to their Ontario-born matches, with no minor illnesses (in either the emergency department or primary care), Years 1 and 2 and Years 3 and 4

© 2026 Wanigaratne S et al. *JAMA Network Open*.

eTable 12. Frequencies for the top 5 *ICD-10* chapters for most responsible diagnosis for ED triage 4 to 5 visits for each exposure group and their matched group

eTable 13. Frequencies for the top 5 *ICD-10* chapters for most responsible diagnosis for ED triage 4 to 5 visits with a family practice sensitive condition for each exposure group and their matched group

eReferences.

This supplemental material has been provided by the authors to give readers additional information about their work.

eFigure 1: Study flow diagram

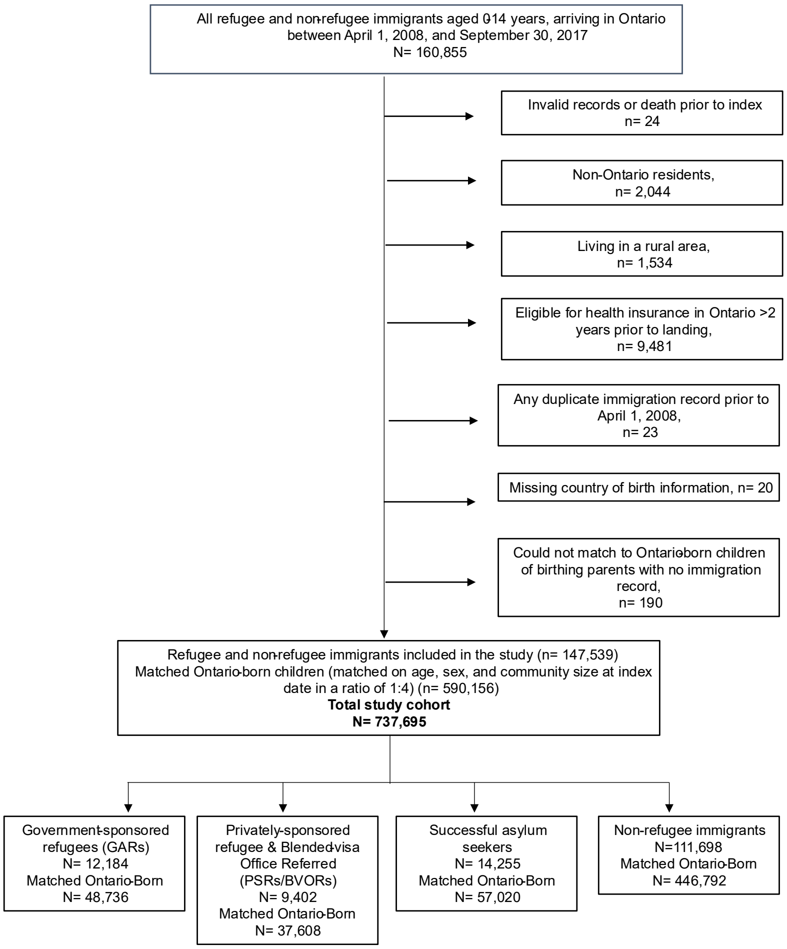

© 2026 Wanigaratne S et al. *JAMA Network Open*.

eTable 1: Description of linked administrative databases

Database	Description
Immigration, Refugees, and Citizenship Canada's Permanent Resident Database	The Ontario portion of the IRCC Permanent Resident Database includes immigration application records for people who initially applied to land in Ontario since 1985. The dataset contains permanent residents' demographic information such as country of citizenship, level of education, mother tongue, and landing date. Overall, 86.4% of immigrants and 92% of refugees in the IRCC database were linked to the healthcare registry, with minimal variability by immigration category, region of birth and minimal differences in characteristics between linked and unlinked people[1].
Ontario Health Insurance Program (OHIP) claims history Database	The OHIP claims database contains information on inpatient and outpatient services provided to Ontario residents eligible for the province's publicly funded health insurance system by fee-for service health care practitioners (primarily physicians) and "shadow billings" for those paid through non-fee-for-service payment plans with the exception of services provided at Community Health Centers (see "CHC").
OHIP Registered Persons Database (RPDB) (healthcare registry)	Basic demographic information (age, sex, location of residence, date of birth, and date of death for deceased individuals) for all individuals issued an Ontario health insurance number. Also indicates the time periods individuals are eligible for health insurance and provides the best-known postal code for each registrant on July 1st of each year which is updated yearly. The RPDB is linkable to all other ICES data holdings using an encrypted health card number.
Postal Code Conversion File (PCCF)+	A conversion template between the six-character postal code and Statistics Canada's standard geographic areas. Through the link between postal codes and standard geographic areas (e.g., dissemination area [DA] which consists of ~400-700 people), the PCCF permits the integration of data from various census derived variables including neighborhood income quintile.
Ontario Marginalization Index (ON-MARG)	ONMARG is a census-based index developed to quantify the degree of marginalization occurring across the province of Ontario. It consists of 4 major dimensions though to underlie the construct of marginalization: households and dwellings (which includes household density), material resources (which includes a combination of income and related factors), age and labour force (vulnerability with respect to age), and racialized and newcomer population (to describe recent immigrants and those who self-identify as a 'visible minority).
National Ambulatory Care Reporting System (NACRS)	NACRS contains administrative, clinical (diagnoses and procedures) and demographic information for all patient visits to hospital- and community-based ambulatory care centres (emergency departments, day surgery units, hemodialysis units, and cancer care clinics) in Ontario. Records are sent from hospitals to CIHI, where they are validated and cleaned before being sent to ICES for use in healthcare administrative research. At ICES, NACRS records are linked with other data sources to identify transitions to other settings, such as inpatient acute care or psychiatric care, and can be linked to other ICES data using the individual's encrypted health card number.
ICES Mother-Baby Linked Database (MOMBABY)	The ICES MOMBABY Database links the DAD inpatient admission records of delivering mothers and their newborns. From 2002 onward, this linkage is performed deterministically using a maternal-newborn chart matching number. Prior to 2002, mothers were linked to their children by matching on the institutions they were admitted, their postal codes, and their admission/discharge dates.
Community Health Centre (CHC)	The CHC dataset provides information on CHC patients and providers that provide care at CHCs. The data is entered into Electronic Medical Records as part of chart records and extracted into a provincial data warehouse, CHC providers do not bill for OHIP therefore data in the CHC is not captured in other datasets.

© 2026 Wanigaratne S et al. *JAMA Network Open*.

Database	Description
Discharge Abstract Database (DAD)	The DAD is compiled by the Canadian Institute for Health Information (CIHI) and contains administrative, clinical (diagnoses and procedures/interventions), demographic, and administrative information for all admissions to acute care hospitals in Ontario. At ICES, consecutive DAD records are linked together to form ‘episodes of care’ among the hospitals to which patients have been transferred after their initial admission.
Same-Day Surgery (SDS) database	The SDS is compiled by the Canadian Institute for Health Information (CIHI) and contains administrative, clinical (diagnoses and procedures), demographic, and administrative information for all patient visits made to day surgery institutions in Ontario.
Client Agency Program Enrollment (CAPE) Database	The CAPE Database is a registry of all patients who have ever been rostered to receive care from a particular physician in Ontario and documents the time period in which a patient was rostered to a specific physician.
ICES Physician Database (IPDB)	The IPDB provides information about all physicians who have practiced in Ontario. The database contains information on demographics (age, gender, year of graduation, school of graduation); specialty (functional and certified); location of practice; and measures of physician activity (billings and workload data)
OHIP Corporate Provider Database (CPDB)	The CPDB provides information on providers/groups eligible to receive payment from OHIP. It contains provider information such as billing number, registration number, demographic information (age, sex), billing specialty, practice location, and group affiliation.

© 2026 Wanigaratne S et al. *JAMA Network Open*.

eTable 2: List of variables, definitions, database, and dates used.

Variable	Data source(s)	Definition
**Exposure**		
Immigration Pathway	- Immigration, Refugees, and Citizenship Canada's (IRCC) Permanent Resident Database<br>- ICES MOMBABY	- Government-sponsored refugees<br>- Privately sponsored refugees and Blended-via Office Referred refugees<br>- Successful asylum-seekers (Protected Persons)<br>- Non- refugee immigrants (economic and family class immigrants)<br>- Ontario-born - persons born in Ontario (in MOMBABY) to a birthing parent without an IRCC record
**Primary outcome**		
Proportion of all minor visits (emergency department + sick visits to primary care) seen in the ED	- National Ambulatory Care Reporting System (NACRS)<br>- Ontario Health Insurance Plan (OHIP)	- NACRS-ED visits of triage score 4 and 5 with a home discharge, excluding visits occurring less than a day after a primary care sick visit.<br>- OHIP – sick visits defined as all primary care codes to family physicians, community medicine and pediatricians and excluding fee codes summarized in Supplementary Table 3.<br>- Proportion using visits described above = NACRS visits/ (NARCS visits + OHIP visits).
**Sensitivity analysis**		
Proportion of all minor visits (defined above) with a Minor ED visit with for a Family Practice Sensitive Condition (FPSC) diagnosis	- National Ambulatory Care Reporting System (NACRS)<br>- Ontario Health Insurance Plan (OHIP)	- ED visits of triage score 4 and 5 with a FPSC diagnosis listed in Supplemental Table 4, with a home discharge, excluding visits occurring less than a day after a primary care sick visit.<br>- OHIP – sick visits defined as all primary care codes to family physicians, community medicine and pediatricians and excluding fee codes summarized in **Supplementary Table 4**.
**Secondary outcomes**		
Routine primary care visits	- Community Health Centres (CHCs)<br>- Ontario Health Insurance Plan (OHIP)	- Primary care sick visits and routine primary care visits to physicians, and nurse practitioners in CHCs walk-in clinics (without a triage score 4+5 ED visit with discharge less than 24 hours after sick visit), see **Supplementary Table 4**
Acute ED visits	- National Ambulatory Care Reporting System (NACRS)	- ED visits of triage score 1-3.
**Post-hoc analysis (among those with no minor ED visits)**		
Routine primary care visits	- Community Health Centres (CHCs)<br>- Ontario Health Insurance Plan (OHIP)	- Primary care sick visits and routine primary care visits to physicians, and nurse practitioners in CHCs walk-in clinics (without a triage score 4+5 ED visit with discharge less than 24 hours after sick visit), see **Supplementary Table 4**.
Acute ED visits	- National Ambulatory Care Reporting System (NACRS)	- ED visits of triage score 1-3.
Acute ED visits that led to hospital admission	- National Ambulatory Care Reporting System (NACRS)	- ED visits of triage score 1-3 resulting in admission to acute care or an inpatient facility.
**Covariates**		

© 2026 Wanigaratne S et al. *JAMA Network Open*.

Variable	Data source(s)	Definition
Year of immigration	- Immigration, Refugees, and Citizenship Canada's (IRCC) Permanent Resident Database	- Year of arrival in Canada as documented in the IRCC database.
Age on index date	- Registered Persons Database (RPDB)	- Age of the individual at index, calculated from the date of birth recorded on the individuals' health card.
Sex on index date	- Registered Persons Database (RPDB)	- Biological sex as recorded in the RPDP database.
Primary care affiliation (measured at index date and 2 years after index date).	- Client Agency Program Enrolment (CAPE)<br>- Corporate Provider Database (CPDB)<br>- Community Health Centres (CHCs)<br>- Ontario Health Insurance Plan (OHIP)	- The primary care access model describes the primary care model recorded for the individual based on rostering and primary care use one year prior to the index date. As in previous studies[2], the primary care access model was assigned in a stepwise approach.<br>o First, all individuals who used a community health centre (CHC) were identified and assigned to the category ***"CHC".***<br>o In a second step, CAPE was used to describe patient rostered to a physician in primary care enrollment groups assigned to categories **"comprehensive"** to describe patients enrolled to capitation-based models, family health teams, or other enrollment groups.<br>o In a third step, the remaining individuals who were not rostered were virtually assigned to the primary care model delivered by the physician who billed the highest amount of money for primary care visits or "**pediatrician**" to describe those enrolled to pediatricians, or "**non-comprehensive**" to describe fee-for-service practices of general practitioner/family physicians who are usually solo-practitioners, or those whose practices do not provide comprehensive services, or walk-in clinics, and does not belong to an enrolment.<br>o If no primary care visit was billed, the individual was assigned to the category: "**no regular provider**".
Major Aggregated Diagnosis Groups (ADGs) (measured at index date and 2 years after index date)	- Ontario Health Insurance Plan (OHIP)<br>- Discharge Abstract Database (DAD)<br>- National Ambulatory Care Reporting System (NACRS)<br>- Same Day Surgery (SDS)	- The ADGs are aggregated diagnostic clusters defined using the Johns Hopkins Adjusted Clinical Groups ACG® case-mix System (version 10)[3] to measure patient co-morbidity. This method groups all patient diagnoses into 32 diagnosis clusters based on the following dimensions:<br>o Duration of the condition (acute, recurrent, or chronic)<br>o Severity of the condition (e.g., minor, and stable versus major and unstable)

© 2026 Wanigaratne S et al. *JAMA Network Open*.

Variable	Data source(s)	Definition
		o Diagnostic certainty (symptoms focusing on diagnostic evaluation versus documented disease focusing on treatment services)<br>o Etiology of the condition (infectious, injury, or other.<br>o Specialty care involvement (medical, surgical, obstetric, hematology, etc.).<br>These clusters were summed and categorized as a categorical yes/no variable.
Material resources quintile (measured at index date and 2 years after index date)	- Ontario Marginalization Index (ONMARG)	- Material resources is a dimension from the Ontario Marginalization Index[4,5] and includes the proportion of:<br>o Proportion of lone-parent families<br>o Proportion aged 25+ without post secondary education.<br>o Proportion receiving government transfer payments.<br>o Proportion aged 15+ who are unemployed.<br>o Proportion of low-income population.<br>o Proportion of dwellings in need of major repair<br>It is categorized in quintiles from Q1 (most resources) to Q5 (least resources). It includes CENSUS data from Statistics Canada at the level of a dissemination area (400-700 inhabitants).

© 2026 Wanigaratne S et al. *JAMA Network Open*.

eTable 3: List of ICD-10 CA diagnosis codes indicating Family Practice Sensitive Conditions

ICD-10-CA codes (main diagnoses)	Description
A740	Chlamydial conjunctivitis
B309	Viral conjunctivitis, unspecified
H100	Mucopurulent conjunctivitis
H101	Acute atopic conjunctivitis
H102	Other acute conjunctivitis
H103	Acute conjunctivitis, unspecified
H104	Chronic conjunctivitis
H105	Blepharoconjunctivitis
H108	Other conjunctivitis
H109	Conjunctivitis, unspecified
H130	Filarial infection of conjunctiva
H131	Conjunctivitis in infectious and parasitic diseases classified elsewhere
H132	Conjunctivitis in other diseases classified elsewhere
H133	Ocular pemphigoid
N300	Acute cystitis
N301	Interstitial cystitis (chronic)
N302	Other chronic cystitis
N303	Trigonitis
N304	Irradiation cystitis
N308	Other cystitis
N309	Cystitis, unspecified
N330	Tuberculous cystitis
N390	Urinary tract infection, site not specified
H650	Acute serous otitis media
H651	Other acute nonsuppurative otitis media
H652	Chronic serous otitis media
H653	Chronic mucoid otitis media
H654	Other chronic nonsuppurative otitis media
H659	Nonsuppurative otitis media, unspecified
H660	Acute suppurative otitis media
H661	Chronic tubotympanic suppurative otitis media
H662	Chronic atticoantral suppurative otitis media
H663	Other chronic suppurative otitis media
H664	Suppurative otitis media, unspecified
H669	Otitis media, unspecified
H670	Otitis media in bacterial diseases classified elsewhere
H671	Otitis media in viral diseases classified elsewhere
H678	Otitis media in other diseases classified elsewhere

© 2026 Wanigaratne S et al. *JAMA Network Open*.

ICD-10-CA codes (main diagnoses)	Description
J00	Acute nasopharyngitis [common cold]
J010	Acute maxillary sinusitis
J011	Acute frontal sinusitis
J012	Acute ethmoidal sinusitis
J013	Acute sphenoidal sinusitis
J014	Acute pansinusitis
J018	Other acute sinusitis
J019	Acute sinusitis, unspecified
J028	Acute pharyngitis due to other specified organisms
J029	Acute pharyngitis, unspecified
J038	Acute tonsillitis due to other specified organisms
J039	Acute tonsillitis, unspecified
J040	Acute laryngitis
J041	Acute tracheitis
J060	Acute laryngopharyngitis
J068	Other acute upper respiratory infections of multiple sites
J069	Acute upper respiratory infection, unspecified
J310	Chronic rhinitis
J311	Chronic nasopharyngitis
J312	Chronic pharyngitis
J320	Chronic maxillary sinusitis
J321	Chronic frontal sinusitis
J322	Chronic ethmoidal sinusitis
J323	Chronic sphenoidal sinusitis
J324	Chronic pansinusitis
J328	Other chronic sinusitis
J329	Chronic sinusitis, unspecified
J350	Chronic tonsillitis
J351	Hypertrophy of tonsils
J352	Hypertrophy of adenoids
J353	Hypertrophy of tonsils with hypertrophy of adenoids
J358	Other chronic diseases of tonsils and adenoids
J359	Chronic disease of tonsils and adenoids, unspecified
J399	Disease of upper respiratory tract, unspecified

© 2026 Wanigaratne S et al. *JAMA Network Open*.

eTable 4: Core Primary Care Services and Routine Primary Care Visits (outside of Community Health Centres)

Fee codes or Diagnosis codes	Description
**Core Primary Care Services**	
A001	MINOR ASSESS. -F.P./G.P.
A002	Family Practice & Practice in General - Enhanced 18 month well baby visit
A003	GEN. ASSESS. -F.P./G.P.
A007	INTERMED.ASSESS/WELL BABY CARE-F.P./G.P./PAED.
A903	GEN/FAM PRACT-PRE-DENTAL/OPER.ASSESS LIMIT 2 PER YEAR/PT
E075	GERIATRIC GENERAL ASSESSMENT PREMIUM
G212	D./T. PROC.-ALLERGY-HYPOSENSITIZATION INJECTION PLUS BASIC
G271	D./T. PROC.-CARDIOV-ANTICOAGULANT SUPERVISION
G372	D./T. PROC.-INJECTIONS-INTRADERMAL/MUSCULAR ETC. EA. ADD.
G373	D./T. PROC.-INJ. INTRADERMAL/MUSC. BASIC FEE (SHICK TEST)
G365	D./T. PROC.-GYNAECOLOGY-PAPANICOLAOU SMEAR
G538	D&T IMMUNIZATION-WITH VISIT, EACH INJECT.
G539	Injection of unspecified agent - sole reason (first injection)
G590	INFLUENZA AGENT +VISIT
G591	Injection of influenza agent - sole reason
K005	INDIVIDUAL CARE PER 1/2 HR
K013	COUNSELLING-ONE OR MORE PEOPLE-PER 1/2HR.
K017	ANNUAL HEALTH EXAM-CHILD AFT. 2ND BIRTHDAY.
P004	OBS.-PRENATAL CARE-MINOR PRENATAL ASSESS.-SUBSEQ.PRENAT.VIS.
K130	Periodic health visit - adolescent
K131	Periodic health visit - adult aged 18 to 64 inclusive
K132	Periodic health visit - adult 65 years of age and older
K030	DIABETIC MANAGEMENT FEE
K080	Minor assessment - Covid, Virtual
K081	Intermediate assessment - Covid, Virtual
K082	Primary mental health care - Covid, Virtual
A261	MINOR ASSESS-PAED.
A268	Pediatrics - Enhanced 18 month well baby visit
K267	ANNUAL HEALTH EXAM-CHILD-AFT. 2ND BIRTHDAY PAED.
K269	ANNUAL HEALTH EXAM-PAEDIATRICS-ADOLESCENT-OFFICE
**Routine Primary Care Visits (Diagnoses Codes in addition to the fee codes above)**	
895	Family Planning: Family planning, contraceptive advice, advice on sterilization or abortion
896	Immunization: Immunization-all types
960	Immunization: Pentavalent (DPT POLIO/ACT HIB)
961	Immunization: DPT Polio
962	Immunization: DT
963	Immunization: MMR (Measles, Mumps, Rubella)
964	Immunization: Hepatitis B
965	Immunization: TD Polio
966	Immunization: TD (Adults and aged 7 years and older)
967	Immunization: Influenza

© 2026 Wanigaratne S et al. *JAMA Network Open*.

Fee codes or Diagnosis codes	Description
968	Immunization: Pneumococcal
969	Immunization: Other Immunization-Not Defined
897	Social, Marital and Family Problems: Economic problems
898	Social, Marital and Family Problems: Marital difficulties
899	Social, Marital and Family Problems: Parent-child problems (e.g., child-abuse, battered child, child neglect)
900	Social, Marital and Family Problems: Problems with aged parents or in-laws
901	Social, Marital and Family Problems: Family disruption, divorce
902	Social, Marital and Family Problems: Educational problems
903	Social, Marital and Family Problems: Illegitimacy
904	Social, Marital and Family Problems: Social maladjustment
905	Social, Marital and Family Problems: Occupational problems, unemployment, difficulty at work
906	Social, Marital and Family Problems: Legal problems, litigation, imprisonment
909	Social, Marital and Family Problems: Other problems of social adjustment
916	Other: Well baby care
917	Other: Annual health examination adolescent/adult Well Vision Care

© 2026 Wanigaratne S et al. *JAMA Network Open*.

eTable 5: Community Health Centre (CHCs) Routine Primary Care Services Visit Types and related Diagnoses Codes

**Routine Primary Care Services (in addition to Z001 routine child health examination)**	
Anonymous HIV service	
Care plan documentation	
Case conference	
Chart Review	
Client care written correspondence	
Client intake/interview	
Counselling regarding breastfeeding	
Cultural ceremony	
Cultural teaching	
Family planning/birth control	
Family/couple counselling	
Foot care	
Forms completion	
Health card registration services	
Individual counselling	
Information provision about community resources	
Interpretation	
Interpreter dispatching	
Medication Reconciliation	
Medication prescription	
Medication renewal	
Mental health care	
Other identification services	
Periodic health examination	
Permanent health card application	
Physical therapy	
Postnatal care	
Prenatal care	
Preventive care	
Psychological assessment	
Recommendation/assistance	
Repeated Assessment Forms Completion (grandfathered)	
Repeated assessment	
Speech/language therapy	
Transportation assistance	
Undefined	
Unknown	
Well baby health examination	
Well baby support	
Written translation of care provided to the patient	
Medication prescription	
Well child health examination	
**ICD-10-CA Diagnoses Codes**	
Z001	Routine child health examination
Z300	General counselling and advice on contraception
Z301	Insertion of (intrauterine) contraceptive device
Z303	Menstrual extraction
Z304	Surveillance of contraceptive drugs
Z305	Surveillance of (intrauterine) contraceptive device

© 2026 Wanigaratne S et al. *JAMA Network Open*.

Z309	Contraceptive management, unspecified
Z315	Genetic counselling
Z319	Procreative management, unspecified
Z349	Supervision of normal pregnancy, unspecified
Z353	Supervision of pregnancy with history of insufficient antenatal care
Z356	Supervision of very young primigravida
Z357	Supervision of high-risk pregnancy due to social problems
Z359	Supervision of high-risk pregnancy, unspecified
Z371	Single stillbirth
Z392	Routine postpartum follow-up
Z412	Routine and ritual circumcision
Z413	Ear piercing

© 2026 Wanigaratne S et al. *JAMA Network Open*.

eTable 6: Characteristics of immigrant children and youth compared to their Ontario-born matches

Characteristics N (% of column population), unless otherwise indicated	GARs N= 12,184	Ontario-Born matches N= 48,736	SD [a]	PSRs/BVORs N= 9,402	Ontario-Born matches N= 37,608	SD [a]	PPs N= 14,255	Ontario-Born matches N= 57,020	SD [a]	NRIs N= 111,698	Ontario-Born matches N= 446,792	SD [a]
**Age at index**												
1-3 years	2,059 (16.9)	8,236 (16.9)	0	1,537 (16.3)	6,148 (16.3)	0	830 (5.8)	3,320 (5.8)	0	20,516 (18.4)	82,064 (18.4)	0
4-6 years	2,711 (22.3)	10,844 (22.3)	0	2,064 (22.0)	8,256 (22.0)	0	3,081 (21.6)	12,324 (21.6)	0	23,706 (21.2)	94,824 (21.2)	0
7-9 years	2,796 (22.9)	11,184 (22.9)	0	2,097 (22.3)	8,388 (22.3)	0	3,539 (24.8)	14,156 (24.8)	0	21,938 (19.6)	87,752 (19.6)	0
10-12 years	2,436 (20.0)	9,744 (20.0)	0	1,882 (20.0)	7,528 (20.0)	0	3,618 (25.4)	14,472 (25.4)	0	21,977 (19.7)	87,908 (19.7)	0
13-15 years	2,178 (17.9)	8,712 (17.9)	0	1,772 (18.8)	7,088 (18.8)	0	3,029 (21.2)	12,116 (21.2)	0	21,419 (19.2)	85,676 (19.2)	0
16+ years	≤5 (0.0)	16 (0.0)	0	50 (0.5)	200 (0.5)	0	158 (1.1)	632 (1.1)	0	2,131 (1.9)	8,524 (1.9)	0
**Sex**												
Female	5,953 (48.9)	23,812 (48.9)	0	4,530 (48.2)	18,120 (48.2)	0	7,010 (49.2)	28,040 (49.2)	0	53,282 (47.7)	213,128 (47.7)	0
Male	6,231 (51.1)	24,924 (51.1)	0	4,872 (51.8)	19,488 (51.8)	0	7,245 (50.8)	28,980 (50.8)	0	58,416 (52.3)	233,664 (52.3)	0
**Material Resources Quintile (at index)**												
Missing	0 (0.0)	26 (0.1)	0.03	1-5[b]	29 (0.1)	0.02	9 (0.1)	35 (0.1)	0	46 (0.0)	290 (0.1)	0.01
1 (most)	254 (2.1)	15,402 (31.6)	**0.86**	51-515[c]	10,691 (28.4)	**0.64**	548 (3.8)	14,883 (26.1)	**0.66**	10,446 (9.4)	108,227 (24.2)	**0.41**
2	401 (3.3)	10,970 (22.5)	**0.60**	801 (8.5)	8,949 (23.8)	**0.42**	736 (5.2)	13,796 (24.2)	**0.56**	13,587 (12.2)	107,132 (24.0)	**0.31**
3	1,483 (12.2)	7,477 (15.3)	0.09	1,143 (12.2)	6,687 (17.8)	**0.16**	1,231 (8.6)	10,847 (19.0)	**0.30**	17,737 (15.9)	88,674 (19.8)	0.10
4	2,380 (19.5)	6,723 (13.8)	**0.15**	1,926 (20.5)	5,539 (14.7)	**0.15**	2,651 (18.6)	8,869 (15.6)	0.08	26,475 (23.7)	74,556 (16.7)	0.18
5 (least)	7,666 (62.9)	8,138 (16.7)	**1.07**	5,016 (53.4)	5,713 (15.2)	**0.88**	9,080 (63.7)	8,590 (15.1)	**1.15**	43,407 (38.9)	67,913 (15.2)	**0.55**
**Material Resources Quintile (at 2 years after index)**												
Missing	45 (0.4)	175 (0.4)	0	29 (0.3)	121 (0.3)	0	58 (0.4)	201 (0.4)	0.01	728 (0.7)	1,434 (0.3)	0.05
1 (most)	321 (2.6)	14,796 (30.4)	**0.81**	498 (5.3)	10,275 (27.3)	**0.62**	702 (4.9)	15,498 (27.2)	**0.64**	13,502 (12.1)	115,162 (25.8)	**0.35**
2	483 (4.0)	11,331 (23.2)	**0.59**	767 (8.2)	9,239 (24.6)	**0.45**	1,050 (7.4)	14,242 (25.0)	**0.49**	17,103 (15.3)	110,805 (24.8)	**0.24**
3	1,170 (9.6)	7,889 (16.2)	**0.20**	1,190 (12.7)	7,068 (18.8)	**0.17**	1,551 (10.9)	10,779 (18.9)	**0.23**	19,488 (17.4)	87,648 (19.6)	0.06
4	2,137 (17.5)	6,693 (13.7)	**0.10**	1,876 (20.0)	5,504 (14.6)	**0.14**	2,762 (19.4)	8,500 (14.9)	**0.12**	24,887 (22.3)	69,171 (15.5)	**0.17**
5 (least)	8,028 (65.9)	7,852 (16.1)	**1.17**	5,042 (53.6)	5,401 (14.4)	**0.91**	8,132 (57.0)	7,800 (13.7)	**1.02**	35,990 (32.2)	62,572 (14.0)	**0.44**
**Major Morbidity (at index)**												
No	10,928 (89.7)	44,926 (92.2)	0.09	8,672 (92.2)	34,611 (92.0)	0.01	13,253(93.0)	52,590 (92.2)	0.03	104,799 (93.8)	410,714 (91.9)	0.07
Yes	1,256 (10.3)	3,810 (7.8)	0.09	730 (7.8)	2,997 (8.0)	0.01	1,002 (7.0)	4,430 (7.8)	0.03	6,899 (6.2)	36,078 (8.1)	0.07
**Major Morbidity (at 2 years after index)**												
No	11,115 (91.2)	44,881 (92.1)	0.03	8,687 (92.4)	34,546 (91.9)	0.02	13,317(93.4)	52,412 (91.9)	0.06	104,449 (93.5)	409,453 (91.6)	0.07
Yes	1,069 (8.8)	3,855 (7.9)	0.03	715 (7.6)	3,062 (8.1)	0.02	938 (6.6)	4,608 (8.1)	0.06	7,249 (6.5)	37,339 (8.4)	0.07
**Primary care affiliation (at index)**												
CHC	2,531 (20.8)	387 (0.8)	**0.68**	768 (8.2)	278 (0.7)	**0.37**	862 (6.0)	239 (0.4)	**0.32**	2,187 (2.0)	2,184 (0.5)	**0.13**
Comprehensive care	4,599 (37.7)	33,012 (67.7)	**0.63**	3,773 (40.1)	24,320 (64.7)	**0.51**	5,642 (39.6)	35,756 (62.7)	**0.48**	51,321 (45.9)	274,505 (61.4)	**0.31**
Pediatrician	411 (3.4)	2,732 (5.6)	**0.11**	202 (2.1)	2,789 (7.4)	**0.25**	380 (2.7)	4,851 (8.5)	**0.26**	2,177 (1.9)	42,305 (9.5)	**0.33**
Other PCP	3,764 (30.9)	6,334 (13.0)	**0.44**	3,533 (37.6)	5,065 (13.5)	**0.58**	4,956 (34.8)	7,543 (13.2)	**0.52**	32,239 (28.9)	64,529 (14.4)	**0.36**
No PCP	879 (7.2)	6,271 (12.9)	**0.19**	1,126 (12.0)	5,156 (13.7)	0.05	2,415 (16.9)	8,631 (15.1)	0.05	23,774 (21.3)	63,269 (14.2)	**0.19**
**Primary care affiliation (at 2 years after index)**												
CHC	1,064 (8.7)	471 (1.0)	**0.37**	479 (5.1)	326 (0.9)	**0.25**	761 (5.3)	388 (0.7)	**0.28**	1,668 (1.5)	2,835 (0.6)	0.08
Comprehensive care	6,935 (56.9)	33,827 (69.4)	**0.26**	5,243 (55.8)	24,995 (66.5)	**0.22**	7,804 (54.7)	36,953 (64.8)	**0.21**	68,532 (61.4)	284,872 (63.8)	0.05
Pediatrician	304 (2.5)	2,075 (4.3)	**0.10**	129 (1.4)	2,124 (5.6)	**0.23**	203 (1.4)	3,914 (6.9)	**0.28**	1,467 (1.3)	33,657 (7.5)	**0.31**

© 2026 Wanigaratne S et al. *JAMA Network Open*.

Characteristics N (% of column population), unless otherwise indicated	GARs N= 12,184	Ontario-Born matches N= 48,736	SD [a]	PSRs/BVORs N= 9,402	Ontario-Born matches N= 37,608	SD [a]	PPs N= 14,255	Ontario-Born matches N= 57,020	SD [a]	NRIs N= 111,698	Ontario-Born matches N= 446,792	SD [a]
Other PCP	2,421 (19.9)	5,451 (11.2)	**0.24**	2,341 (24.9)	4,400 (11.7)	**0.35**	3,049 (21.4)	6,767 (11.9)	**0.26**	18,890 (16.9)	56,941 (12.7)	**0.12**
No PCP	1,460 (12.0)	6,912 (14.2)	0.07	1,210 (12.9)	5,763 (15.3)	0.07	2,438 (17.1)	8,998 (15.8)	0.04	21,141 (18.9)	68,487 (15.3)	**0.10**
**Arrival year**												
2008 – 2010	2,353 (19.3)	NA	NA	1,669 (17.8)	NA	NA	4,255 (29.8)	NA	NA	43,520 (39.0)	NA	NA
2011 – 2014	2,781 (22.8)	NA	NA	2,030 (21.6)	NA	NA	6,230 (43.7)	NA	NA	44,340 (39.7)	NA	NA
2015 - 2017	7,050 (57.9)	NA	NA	5,703 (60.6)	NA	NA	3,770 (26.5)	NA	NA	23,838 (21.3)	NA	NA
**Official language ability at arrival**												
English	1,202 (9.9)	NA	NA	1,947 (20.7)	NA	NA	7,836 (55.0)	NA	NA	52,503 (47.0)	NA	NA
French	118 (1.0)	NA	NA	66 (0.7)	NA	NA	946 (6.6)	NA	NA	1,307 (1.2)	NA	NA
Bilingual	42 (0.3)	NA	NA	29 (0.3)	NA	NA	458 (3.2)	NA	NA	1,294 (1.2)	NA	NA
Neither	10,481 (86.0)	NA	NA	7,190 (76.5)	NA	NA	4,862 (34.1)	NA	NA	55,868 (50.0)	NA	NA
Missing	341 (2.8)	NA	NA	170 (1.8)	NA	NA	153 (1.1)	NA	NA	726 (0.6)	NA	NA
**Healthcare eligibility prior to arrival date (possible history of being a temporary resident)**												
Yes	287 (2.4)	NA	NA	107 (1.1)	NA	NA	6,267 (44.0)	NA	NA	4,988 (4.5)	NA	NA
**Age at Arrival**												
0 – 3 years	2,926 (24.1)	NA	NA	2,281 (24.3)	NA	NA	1,168 (8.2)	NA	NA	31,237 (28.0)	NA	NA
4 – 9 years	5,492 (45.1)	NA	NA	4,112 (43.7)	NA	NA	7,041 (49.4)	NA	NA	44,625 (40.0)	NA	NA
10 – 15 years	3,746 (30.8)	NA	NA	3,009 (32.0)	NA	NA	6,046 (42.4)	NA	NA	35,836 (32.0)	NA	NA
**Birth region**												
Central Africa	374 (3.1)	NA	NA	57 (0.6)	NA	NA	366 (2.6)	NA	NA	425 (0.4)	NA	NA
Western Africa	87 (0.7)	NA	NA	37 (0.4)	NA	NA	878 (6.2)	NA	NA	2,600 (2.3)	NA	NA
East Africa	1,280 (10.5)	NA	NA	848 (9.0)	NA	NA	1,406 (9.9)	NA	NA	1,347 (1.2)	NA	NA
Southern Africa	86 (0.7)	NA	NA	68 (0.7)	NA	NA	87 (0.6)	NA	NA	402 (0.4)	NA	NA
Middle East	8,329 (68.4)	NA	NA	6,420 (68.3)	NA	NA	1,111 (7.8)	NA	NA	17,517 (15.7)	NA	NA
North Africa	220 (1.8)	NA	NA	310 (3.3)	NA	NA	253 (1.8)	NA	NA	4,060 (3.6)	NA	NA
Central America	1-5[b]	NA	NA	1-5[b]	NA	NA	506 (3.5)	NA	NA	1,135 (1.0)	NA	NA
South America	146 (1.2)	NA	NA	63 (0.7)	NA	NA	1,032 (7.2)	NA	NA	2,627 (2.4)	NA	NA
Caribbean	9 (0.1)	NA	NA	1-5[b]	NA	NA	1,183 (8.3)	NA	NA	5,350 (4.8)	NA	NA
North America	1-5[b]	NA	NA	19 (0.2)	NA	NA	2,038 (14.3)	NA	NA	6,209 (5.6)	NA	NA
East Asia	1-5[b]	NA	NA	1-5[b]	NA	NA	1,263 (8.9)	NA	NA	9,697 (8.7)	NA	NA
Australasia & Oceania & Asia Unspecified	0	.NA	NA	0	NA	NA	1-5[b]	NA	NA	492 (0.4)	NA	NA
Southeast Asia	455 (3.7)	NA	NA	147 (1.6)	NA	NA	48 (0.3)	NA	NA	19,116 (17.1)	NA	NA
South Asia	808 (6.6)	NA	NA	1,117 (11.9)	NA	NA	2,970 (20.8)	NA	NA	31,151 (27.9)	NA	NA
Eastern Europe	141 (1.2)	NA	NA	28 (0.3)	NA	NA	679 (4.8)	NA	NA	4,259 (3.8)	NA	NA
Europe other	242 (2.0)	NA	NA	276 (2.9)	NA	NA	430-434[c]	NA	NA	5,311 (4.8)	NA	NA

Abbreviation: N/A, not applicable.

[a] SD < 0.1 (bolded values) indicates variable is balanced between immigrant group and matched Ontario-born population. The SD is estimated only for variables available for both immigrants and the Ontario-born population.

[b] Small cells (<6) suppressed in accordance with ICES policy.

[c] Non-missing data reported as ranges without percentage to reduce risk of re-identification in accordance with ICES policy.

© 2026 Wanigaratne S et al. *JAMA Network Open*.

eTable 7: Characteristics 2 years after index of immigrant children and youth arriving in Ontario 2008-2017 and their Ontario-born matches with at least 1 minor illness visit to primary care or the emergency department in the second 2 years after the index

Characteristics N (% of column population)	GARs N= 9,015	Ontario-Born matches N= 24,293	SD[a]	PSRs/BVORs N= 6,602	Ontario-Born matches N= 17,614	SD[a]	PPs N= 10,383	Ontario-Born matches N= 30,144	SD[a]	NRIs N= 75,975	Ontario-Born matches N= 221,992	SD[a]
**Age**												
1-3 years	453 (5.0)	1,248 (5.1)	0.01	253 (3.8)	674 (3.8)	0	38 (0.4)	116 (0.4)	0	6,794 (3.1)	2,305 (3.0)	0
4-6 years	1,786 (19.8)	4,799 (19.8)	0	1,382 (20.9)	3,688 (20.9)	0	1,306 (12.6)	3,754 (12.5)	0	54,312 (24.5)	18,606 (24.5)	0
7-9 years	2,016 (22.4)	5,182 (21.3)	0.02	1,427 (21.6)	3,614 (20.5)	0.03	2,447 (23.6)	6,809 (22.6)	0.02	43,146 (19.4)	15,425 (20.3)	0.02
10-12 years	1,912 (21.2)	4,959 (20.4)	0.02	1,386 (21.0)	3,568 (20.3)	0.02	2,537 (24.4)	7,185 (23.8)	0.01	41,165 (18.5)	14,450 (19.0)	0.01
13-15 years	1,733 (19.2)	4,787 (19.7)	0.01	1,278 (19.4)	3,464 (19.7)	0.01	2,650 (25.5)	7,894 (26.2)	0.02	41,883 (18.9)	14,102 (18.6)	0.01
16+ years	1,115 (12.4)	3,318 (13.7)	0.04	876 (13.3)	2,606 (14.8)	0.04	1,405 (13.5)	4,386 (14.6)	0.03	34,692 (15.6)	11,087 (14.6)	0.03
**Sex**												
Female	4,437 (49.2)	12,191 (50.2)	0.02	3,238 (49.0)	8,890 (50.5)	0.03	5,184 (49.9)	15,435 (51.2)	0.03	36,531 (48.1)	108,584 (48.9)	0.02
Male	4,578 (50.8)	12,102 (49.8)	0.02	3,364 (51.0)	8,724 (49.5)	0.03	5,199 (50.1)	14,709 (48.8)	0.03	39,444 (51.9)	113,408 (51.1)	0.02
**Material Resources Quintile**												
1 (most)	236 (2.6)	7,486 (30.8)	**0.82**	324 (4.9)	4,801 (27.3)	**0.64**	572 (5.5)	8,052 (26.7)	**0.60**	9,309 (12.3)	56,464 (25.4)	**0.34**
2	376 (4.2)	5,430 (22.4)	**0.56**	509 (7.7)	4,202 (23.9)	**0.45**	739 (7.1)	7,541 (25.0)	**0.50**	11,370 (15.0)	54,799 (24.7)	**0.25**
3	900 (10.0)	3,786 (15.6)	**0.17**	747 (11.3)	3,100 (17.6)	**0.18**	1,101 (10.6)	5,617 (18.6)	**0.23**	12,824 (16.9)	43,054 (19.4)	0.07
4	1,616 (17.9)	3,143 (12.9)	**0.14**	1,284 (19.4)	2,504 (14.2)	**0.14**	1,993 (19.2)	4,548 (15.1)	**0.11**	16,639 (21.9)	34,614 (15.6)	**0.16**
5 (least)	5,524 (61.3)	3,735 (15.4)	**1.07**	3,410 (51.7)	2,437 (13.8)	**0.88**	5,763 (55.5)	4,139 (13.7)	**0.98**	24,195 (31.8)	30,792 (13.9)	**0.44**
Missing	363 (4.0)	713 (2.9)	0.06	328 (5.0)	570 (3.2)	0.09	215 (2.1)	247 (0.8)	0.10	1,638 (2.2)	2,269 (1.0)	0.09
**Major Morbidity**												
No	8,162 (90.5)	22,170 (91.3)	0.03	6,054 (91.7)	15,991 (90.8)	0.03	9,623 (92.7)	27,475 (91.1)	0.06	70,137 (92.3)	201,643 (90.8)	0.05
Yes	853 (9.5)	2,123 (8.7)	0.03	548 (8.3)	1,623 (9.2)	0.03	760 (7.3)	2,669 (8.9)	0.06	5,838 (7.7)	20,349 (9.2)	0.05
**Primary care affiliation**												
CHC	803 (8.9)	294 (1.2)	**0.36**	350 (5.3)	179 (1.0)	**0.25**	588 (5.7)	244 (0.8)	**0.28**	1,312 (1.7)	1,686 (0.8)	0.09
Comprehensive care	5,500 (61.0)	17,983 (74.0)	**0.28**	3,778 (57.2)	12,602 (71.5)	**0.30**	6,008 (57.9)	21,128 (70.1)	**0.26**	51,276 (67.5)	153,442 (69.1)	0.04
Pediatrician	148 (1.6)	877 (3.6)	**0.12**	71 (1.1)	871 (4.9)	**0.23**	140 (1.3)	1,816 (6.0)	**0.25**	850 (1.1)	14,284 (6.4)	0.28
Other PCP	1,873 (20.8)	3,168 (13.0)	**0.21**	1,798 (27.2)	2,387 (13.6)	**0.34**	2,407 (23.2)	4,098 (13.6)	**0.25**	14,532 (19.1)	32,301 (14.6)	**0.12**
No PCP	691 (7.7)	1,971 (8.1)	0.02	605 (9.2)	1,575 (8.9)	0.01	1,240 (11.9)	2,858 (9.5)	0.08	8,005 (10.5)	20,279 (9.1)	0.05

© 2026 Wanigaratne S et al. *JAMA Network Open*.

eTable 8: Modeling the difference in the mean percentage of all minor illness visits seen in the ED (among those who have at least 1 minor illness) and 95% CI between each immigrant group and their Ontario-born matches.

	Year 1 & 2				Year 3 & 4			
	Model 1	Model 2	Model 3	Model 4	Model 1	Model 2	Model 3	Model 4
**Government Assisted Refugees (GARs): N=10,211 vs. Ontario-Born matches: N=31,017**								
	**Matching ratios: 1:4= 34.0%; 1:3= 40.8%; 1:2 =20.1%; 1:1= 5.1%**				**Matching ratios: 1:4= 23.2%; 1:3= 35.9%; 1:2= 28.2%; 1:1= 12.7%**			
**GARs** (ref= Ontario-born match)	-3.88 (-4.31, -3.45)	-3.89 (-4.32, -3.46)	-5.10 (-5.63, -4.57)	-5.11 (-5.66, -4.55)	-3.74 (-4.26, -3.23)	-3.75 (-4.26, -3.24)	-4.54 (-5.18, -3.89)	-4.46 (-5.11, -3.81)
**Major ACG** (ref=No) *****	NA	0.36 (-0.37, 1.1)	0.32 (-0.41, 1.05)	0.48 (-0.25, 1.20)	NA	0.56 (-0.33, 1.46)	0.57 (-0.33, 1.46)	0.75 (-0.14, 1.64)
**Material Resources Quintile** (ref= 1, most resources)**[a]**								
2	NA	NA	-0.09 (-0.77, 0.59)	-0.14 (-0.82, 0.53)	NA	NA	-1.48 (-2.3, -0.65)	-1.5 (-2.31, -0.68)
3	NA	NA	-0.42 (-1.13, 0.28)	-0.34 (-1.04, 0.36)	NA	NA	-0.49 (-1.38, 0.4)	-0.57 (-1.45, 0.32)
4	NA	NA	0.6 (-0.13, 1.32)	0.71 (-0.01, 1.43)	NA	NA	-0.44 (-1.34, 0.46)	-0.47 (-1.36, 0.43)
5 (least)	NA	NA	2.48 (1.76, 3.20)	2.39 (1.68, 3.11)	NA	NA	0.95 (0.08, 1.81)	0.77 (-0.1, 1.63)
**Primary care affiliation** (ref=comprehensive)[a]								
CHC	NA	NA	NA	2.57 (1.63, 3.51)	NA	NA	NA	2.3 (0.86, 3.75)
Pediatrician	NA	NA	NA	7.04 (5.52, 8.56)	NA	NA	NA	6.32 (4.35, 8.29)
Other PCP	NA	NA	NA	-0.26 (-0.76, 0.25)	NA	NA	NA	-0.1 (-0.75, 0.55)
No PCP	NA	NA	NA	9.13 (7.92, 10.33)	NA	NA	NA	9.33 (8, 10.66)
**Privately Sponsored Refugees/Blended Visa Office Referred Refugees (PSRs/BVORs): N=7,810 vs. Ontario-born matches: N=23,467**								
	**Matching ratios: 1:4= 33.0%; 1:3= 40.1%; 1:2= 21.4%; 1:1= 5.6%**				**Matching ratios: 1:4= 22.1%; 1:3= 36.1%; 1:2= 28.4%; 1:1= 13.5%**			
**PSRs/BVORs)** (ref= Ontario-born match)	-4.79 (-5.26, -4.32)	-4.79 (-5.26, -4.32)	-5.43 (-5.98, -4.88)	-5.24 (-5.8, -4.67)	-3.78 (-4.38, -3.18)	-3.78 (-4.38, -3.18)	-4.26 (-4.97, -3.56)	-4.05 (-4.75, -3.34)
**Major ACG** (ref= No)*****	NA	0.05 (-0.8, 0.89)	0.07 (-0.78, 0.91)	0.25 (-0.58, 1.07)	NA	0.33 (-0.71, 1.36)	0.32 (-0.71, 1.36)	0.5 (-0.53, 1.53)
**Material Resources Quintile** (ref = 1, most resources)**[a]**								

© 2026 Wanigaratne S et al. *JAMA Network Open*.

	Year 1 & 2				Year 3 & 4			
	**Model 1**	**Model 2**	**Model 3**	**Model 4**	**Model 1**	**Model 2**	**Model 3**	**Model 4**
2	NA	NA	0.28 (-0.49, 1.05)	0.26 (-0.5, 1.03)	NA	NA	-1 (-1.95, -0.05)	-1 (-1.94, -0.06)
3	NA	NA	0.24 (-0.55, 1.04)	0.36 (-0.43, 1.14)	NA	NA	-0.75 (-1.75, 0.25)	-0.68 (-1.67, 0.31)
4	NA	NA	1.1 (0.26, 1.93)	1.14 (0.32, 1.96)	NA	NA	-0.43 (-1.46, 0.59)	-0.32 (-1.34, 0.7)
5	NA	NA	1.65 (0.85, 2.46)	1.72 (0.92, 2.51)	NA	NA	0.67 (-0.33, 1.68)	0.75 (-0.26, 1.75)
**Primary care affiliation** (ref= comprehensive)[a]								
CHC	NA	NA	NA	2.57 (1.16, 3.98)	NA	NA	NA	-0.06 (-1.79, 1.66)
Pediatrician	NA	NA	NA	7.42 (5.86, 8.98)	NA	NA	NA	5.1 (3.13, 7.07)
Other PCP	NA	NA	NA	-0.71 (-1.25, -0.17)	NA	NA	NA	-0.43 (-1.16, 0.29)
No PCP	NA	NA	NA	8.48 (7.23, 9.72)	NA	NA	NA	7.44 (6.02, 8.86)
**Successful Asylum Seekers or Protected Persons (PPs): N=11,540 vs. Ontario-born matches: N=34,778**								
	**Matching ratios: 1:4= 32.7%; 1:3= 41.1%; 1:2= 21.0%; 1:1= 5.2%**				**Matching ratios: 1:4= 28.9%; 1:3= 40.1%; 1:2= 23.4%; 1:1= 7.6%**			
**Asylum seekers** (ref= Ontario-born match)	-3.34(-3.74, -2.94)	-3.34 (-3.74, -2.94)	-3.18(-3.65, -2.7)	-3.37 (-3.86, -2.87)	-2.92 (-3.38, -2.46)	-2.92 (-3.38, -2.46)	-2.71 (-3.24, -2.19)	-2.63 (-3.17, -2.10)
**Major ACG** (ref=No)*	NA	-0.08 (-0.79, 0.63)	-0.08 (-0.79, 0.63)	0.04 (-0.66, 0.74)	NA	0.06 (-0.71, 0.83)	0.06(-0.72, 0.83)	0.14 (-0.62, 0.90)
**Material resources Quintile** (ref= 1, most resources)[a]								
2	NA	NA	-0.97 (-1.64, -0.3)	-1 (-1.66, -0.33)	NA	NA	-0.04 (-0.75, 0.68)	0.04 (-0.67, 0.75)
3	NA	NA	-1.05 (-1.74, -0.35)	-1.01 (-1.69, -0.32)	NA	NA	-0.46 (-1.19, 0.28)	-0.24 (-0.97, 0.49)
4	NA	NA	-1.36 (-2.05, -0.68)	-1.19 (-1.87, -0.52)	NA	NA	-1.34 (-2.07, -0.6)	-1.11 (-1.83, -0.38)
5 (least)	NA	NA	-0.87 (-1.53, -0.2)	-0.76 (-1.41, -0.1)	NA	NA	-0.45 (-1.17, 0.27)	-0.24 (-0.96, 0.47)
**Primary care affiliation** (ref = comprehensive)[a]								
CHC	NA	NA	NA	1.23 (-0.02, 2.49)	NA	NA	NA	0.51 (-0.83, 1.85)

© 2026 Wanigaratne S et al. *JAMA Network Open*.

	Year 1 & 2				Year 3 & 4			
	**Model 1**	**Model 2**	**Model 3**	**Model 4**	**Model 1**	**Model 2**	**Model 3**	**Model 4**
Pediatrics	NA	NA	NA	7.07 (5.93, 8.22)	NA	NA	NA	7.18 (5.76, 8.61)
Other PCP	NA	NA	NA	0.38 (-0.07, 0.83)	NA	NA	NA	-0.08 (-0.64, 0.48)
No PCP	NA	NA	NA	8.75 (7.81, 9.69)	NA	NA	NA	7.06 (6.06, 8.05)
**Non-Refugee Immigrants (NRIs): N=83,537 vs. Ontario-born matches: N=256, 237**								
	**Matching ratios: 1:4= 34.6%; 1:3= 41.7%; 1:2= 19.6%; 1:1= 4.1%**				**Matching ratios: 1:4= 29.9%; 1:3= 39.8%; 1:2= 22.8%; 1:1= 7.4%**			
**Non-refugee immigrants** (ref= Ontario-born match)	-4.45 (-4.59, -4.31)	-4.44 (-4.58, -4.3)	-4.19 (-4.34, -4.04)	-4.24 (-4.4, -4.09)	-4.14 (-4.3, -3.98)	-4.13 (-4.29, -3.98)	-3.99 (-4.16, -3.83)	-3.78 (-3.95, -3.62)
**Major ACG** (ref=No) *	NA	0.4 (0.14, 0.66)	0.4 (0.13, 0.66)	0.55 (0.29, 0.81)	NA	0.36 (0.08, 0.64)	0.35 (0.07, 0.63)	0.51 (0.23, 0.79)
**Material resources Quintile,** (ref= 1, most resources)[a]								
2	NA	NA	-0.74 (-0.98, -0.49)	-0.7 (-0.94, -0.46)	NA	NA	-0.55 (-0.79, -0.3)	-0.45 (-0.7, -0.21)
3	NA	NA	-1.64 (-1.88, -1.4)	-1.53 (-1.77, -1.29)	NA	NA	-1.01 (-1.26, -0.76)	-0.86 (-1.11, -0.61)
4	NA	NA	-1.81 (-2.05, -1.57)	-1.59 (-1.83, -1.35)	NA	NA	-1.36 (-1.62, -1.11)	-1.16 (-1.41, -0.9)
5	NA	NA	-1.22 (-1.46, -0.98)	-0.9 (-1.14, -0.67)	NA	NA	-0.7 (-0.96, -0.44)	-0.5 (-0.76, -0.24)
**Primary care affiliation** (ref = comprehensive)[a]								
CHC	NA	NA	NA	0.71 (0.11, 1.3)	NA	NA	NA	0.29 (-0.39, 0.97)
Pediatrician	NA	NA	NA	7.84 (7.41, 8.26)	NA	NA	NA	6.53 (6.03, 7.02)
Other PCP	NA	NA	NA	0.18 (0.02, 0.34)	NA	NA	NA	-0.11 (-0.31, 0.09)
No PCP	NA	NA	NA	7.91 (7.57, 8.25)	NA	NA	NA	7.41 (7.04, 7.79)

Abbreviation: NA, not available

Model 1= Unadjusted model; **Model 2**= adjusted for ACG; **Model 3**: adjusted for ACGs, material resources; **Model 4**= adjusted for ACG, material resources, and primary care affiliation.

[a]For years 1 & 2, measured at beginning of year 1/index (a year after healthcare eligibility). For years 3 & 4, measured at beginning of year 3 (two years after index).

© 2026 Wanigaratne S et al. *JAMA Network Open*.

eTable 9: Modelling the average difference in the proportion of all minor visits seen in the ED with a Family Practice Sensitive Condition (among those who have at least 1 minor visit) and 95% CI between each immigrant group and their Ontario-born matches.

	Year 1 & 2				Year 3 & 4			
	**Model 1**	**Model 2**	**Model 3**	**Model 4**	**Model 1**	**Model 2**	**Model 4**	**Model 4**
**Government Assisted Refugees (GARs): N=10,211 vs. Ontario-Born matches: N=31,017**								
	**Matching ratios: 1:4= 34.0%; 1:3= 40.8%; 1:2 =20.1%; 1:1= 5.1%**				**Matching ratios: 1:4= 23.2%; 1:3= 35.9%; 1:2= 28.2%; 1:1= 12.7%**			
**GARs** (ref= Ontario-born match)	-0.74 (-1.87, 0.39)	-0.76 (-1.88, 0.37)	-3.03 (-4.4, -1.66)	-3.32 (-4.85, -1.79)	-0.47 (-1.51, 0.57)	-0.47 (-1.51, 0.57)	-1.41 (-2.6, -0.22)	-1.4 (-2.58, -0.22)
**Major ACG** (ref=No)[a]	NA	0.67 (-1.04, 2.38)	0.5 (-1.2, 2.2)	0.51 (-1.19, 2.22)	NA	-0.08 (-1.35, 1.18)	-0.07 (-1.33, 1.2)	-0.08 (-1.34, 1.19)
**Material Resources Quintile** (ref = 1, most resources)[a]								
2	NA	NA	0.65 (-0.55,1.84)	0.67 (-0.53, 1.87)	NA	NA	0.65 (-0.65, 1.94)	0.64 (-0.65, 1.94)
3	NA	NA	2.03 (0.63, 3.42)	2.12 (0.73, 3.52)	NA	NA	1.32 (-0.06, 2.7)	1.31 (-0.07, 2.7)
4	NA	NA	2.98 (1.46, 4.49)	3.02 (1.51, 4.53)	NA	NA	1.15 (-0.21, 2.52)	1.2 (-0.18, 2.57)
5 (least)	NA	NA	5.05 (3.57, 6.53)	5.13 (3.66, 6.59)	NA	NA	2.25 (0.92, 3.59)	2.26 (0.92, 3.6)
**Primary care affiliation** (ref =comprehensive)[a]								
CHC	NA	NA	NA	1.54 (-0.73, 3.81)	NA	NA	NA	-0.05 (-2.28, 2.18)
Pediatrician	NA	NA	NA	4.14 (1.43, 6.85)	NA	NA	NA	2.82 (-0.37, 6.01)
Other PCP	NA	NA	NA	0.49 (-0.93, 1.92)	NA	NA	NA	0.24 (-1.16, 1.64)
No PCP	NA	NA	NA	3.21 (1.29, 5.14)	NA	NA	NA	0.76 (-1.03, 2.56)
**Privately Sponsored Refugees/Blended Visa Office Referred Refugees (PSRs/BVORs): N=7,810 vs. Ontario-born matches: N=23,467**								
	**Matching ratios: 1:4= 33.0%; 1:3= 40.1%; 1:2= 21.4%; 1:1= 5.6%**				**Matching ratios: 1:4= 22.1%; 1:3= 36.1%; 1:2= 28.4%; 1:1= 13.5%**			
**PSRs/BVORs)** (ref= Ontario-born match)	-1.13 (-2.42, 0.16)	-1.13 (-2.42, 0.16)	-2.61 (-4.06, -1.15)	-2.38 (-3.85, -0.91)	1.25 (-0.24, 2.74)	1.27 (-0.22, 2.76)	0.41 (-1.31, 2.13)	0.85 (-0.93, 2.63)
**Major ACG** (ref=No)[a]	NA	0.67 (-1.16, 2.49)	0.57 (-1.26, 2.39)	0.61 (-1.21, 2.43)	NA	0.54 (-0.97, 2.05)	0.49 (-1.02, 2)	0.69 (-0.83, 2.22)
**Material Resources Quintile** (ref = 1, most resources)[a]								

© 2026 Wanigaratne S et al. *JAMA Network Open*.

	Year 1 & 2				Year 3 & 4			
	**Model 1**	**Model 2**	**Model 3**	**Model 4**	**Model 1**	**Model 2**	**Model 4**	**Model 4**
2	NA	NA	0.55 (-0.93, 2.04)	0.57 (-0.92, 2.05)	NA	NA	0.03 (-1.25, 1.3)	0.12 (-1.19, 1.43)
3	NA	NA	2.3 (0.75, 3.85)	2.3 (0.76, 3.85)	NA	NA	1.45 (-0.13, 3.03)	1.59 (-0.01, 3.18)
4	NA	NA	4.05 (2.2, 5.89)	4.04 (2.19, 5.88)	NA	NA	1.04 (-0.7, 2.79)	1.13 (-0.64, 2.9)
5	NA	NA	3.88 (2.11, 5.66)	3.94 (2.16, 5.71)	NA	NA	2.34 (0.56, 4.13)	2.39 (0.6, 4.18)
**Primary care affiliation** (ref = comprehensive)[a]								
CHC	NA	NA	NA	0.09 (-2.43, 2.62)	NA	NA	NA	-2.63 (-4.96, -0.3)
Pediatrician	NA	NA	NA	2.08 (-0.49, 4.65)	NA	NA	NA	1.81 (-1.16, 4.78)
Other PCP	NA	NA	NA	-0.58 (-1.87, 0.72)	NA	NA	NA	-1.5 (-2.57, -0.43)
No PCP	NA	NA	NA	4.25 (1.94, 6.56)	NA	NA	NA	3.24 (0.26, 6.23)
**Successful Asylum Seekers or Protected Persons (PPs): N=11,540 vs. Ontario-born matches N=34,778**								
	**Matching ratios: 1:4= 32.7%; 1:3= 41.1%; 1:2= 21.0%; 1:1= 5.2%**				**Matching ratios: 1:4= 28.9%; 1:3= 40.1%; 1:2= 23.4%; 1:1= 7.6%**			
**Asylum seekers** (ref= Ontario-born match)	0.36 (-0.6, 1.33)	0.36 (-0.6, 1.33)	-0.54 (-1.81, 0.72)	-0.62 (-1.94, 0.71)	0.03 (-0.74, 0.79)	0.02 (-0.74, 0.79)	-0.38 (-1.26, 0.5)	-0.48 (-1.37, 0.4)
**Major ACG (ref=No)[a]**	NA	0.85 (-0.64, 2.34)	0.84 (-0.65, 2.33)	0.86 (-0.64, 2.35)	NA	-0.25 (-1.08, 0.57)	-0.24 (-1.06, 0.59)	-0.21 (-1.04, 0.61)
**Material resources Quintile** (ref = 1, most resources)[a]								
2	NA	NA	-0.66 (-1.81, 0.48)	-0.6 (-1.75, 0.55)	NA	NA	0.14 (-0.75, 1.02)	0.19 (-0.69, 1.07)
3	NA	NA	0 (-1.21, 1.2)	0.12 (-1.08, 1.33)	NA	NA	1.05 (-0.02, 2.12)	1.11 (0.04, 2.18)
4	NA	NA	0.52 (-0.76, 1.8)	0.72 (-0.57, 2.01)	NA	NA	0.01 (-0.92, 0.94)	0.04 (-0.89, 0.97)
5 (least)	NA	NA	1.62 (0.14, 3.1)	1.83 (0.34, 3.33)	NA	NA	1.27 (0.2, 2.34)	1.27 (0.21, 2.33)
**Primary care affiliation** (ref = comprehensive)[a]								
CHC	NA	NA	NA	2.63 (-0.85, 6.11)	NA	NA	NA	2.34 (-0.51, 5.19)

© 2026 Wanigaratne S et al. *JAMA Network Open*.

	Year 1 & 2				Year 3 & 4			
	**Model 1**	**Model 2**	**Model 3**	**Model 4**	**Model 1**	**Model 2**	**Model 4**	**Model 4**
Pediatrics	NA	NA	NA	4.43 (2.35, 6.51)	NA	NA	NA	1.61 (-0.29, 3.52)
Other PCP	NA	NA	NA	-0.16 (-1.07, 0.75)	NA	NA	NA	-0.03 (-0.86, 0.81)
No PCP	NA	NA	NA	2.2 (0.52, 3.87)	NA	NA	NA	1.72 (0.3, 3.13)
**Non-Refugee Immigrants (NRIs): N=83,537 vs. Ontario-born matches: N=256, 237**								
	**Matching ratios: 1:4= 34.6%; 1:3= 41.7%; 1:2= 19.6%; 1:1= 4.1%**				**Matching ratios: 1:4= 29.9%; 1:3= 39.8%; 1:2= 22.8%; 1:1= 7.4%**			
**Non-refugee immigrants** (ref= Ontario-born match)	-0.99 (-1.36, -0.63)	-0.98 (-1.35, -0.61)	-1.35 (-1.74, -0.96)	-1.3 (-1.69, -0.9)	-0.68 (-1.01, -0.36)	-0.68 (-1.01, -0.35)	-0.98 (-1.32, -0.65)	-0.9 (-1.23, -0.56)
**Major ACG** (ref=No)[a]	NA	1.49 (0.9, 2.08)	1.47 (0.89, 2.06)	1.49 (0.9, 2.07)	NA	0.49 (0.05, 0.92)	0.48 (0.05, 0.92)	0.51 (0.07, 0.94)
**Material resources Quintile,** (ref=1, most resources)[a]								
2	NA	NA	0.61 (0.16, 1.06)	0.66 (0.21, 1.11)	NA	NA	0.33 (-0.03, 0.7)	0.38 (0.01, 0.74)
3	NA	NA	0.15 (-0.31, 0.61)	0.24 (-0.22, 0.7)	NA	NA	0.53 (0.13, 0.93)	0.59 (0.19, 0.99)
4	NA	NA	0.87 (0.38, 1.37)	1 (0.5, 1.49)	NA	NA	1.05 (0.6, 1.49)	1.11 (0.66, 1.56)
5	NA	NA	1.73 (1.23, 2.24)	1.94 (1.43, 2.45)	NA	NA	1.79 (1.33, 2.25)	1.85 (1.39, 2.32)
**Primary care affiliation** (ref = comprehensive)[a]								
CHC	NA	NA	NA	-0.09 (-1.28, 1.09)	NA	NA	NA	0.36 (-0.91, 1.62)
Pediatrician	NA	NA	NA	3.83 (3.07, 4.59)	NA	NA	NA	2 (1.18, 2.82)
Other PCP	NA	NA	NA	0.34 (-0.04, 0.71)	NA	NA	NA	0.08 (-0.27, 0.43)
No PCP	NA	NA	NA	3.53 (2.9, 4.16)	NA	NA	NA	2.17 (1.5, 2.85)

Abbreviation: NA, not available

Model 1= Unadjusted model; **Model 2**= adjusted for ACG; **Model 3**: adjusted for ACGs, material resources; **Model 4**= adjusted for ACG, material resources, and primary care affiliation.

[a]For years 1 & 2, measured at beginning of year 1 (index date, a year after healthcare eligibility). For years 3 & 4, measured at beginning of year 3 (two years after index).

© 2026 Wanigaratne S et al. *JAMA Network Open*.

eTable 10: Primary outcome, sensitivity outcome and complementary measures among immigrant children and youth compared to their Ontario-born matches, with at least 1 minor illness seen in the emergency department or primary care, in Years 1 and 2 and Years 3 and 4

Immigrant Groups and Ontario-born matches (1:4)	GARs N= 12,184	Ontario-Born N= 48,736	PSRs/BVORs N= 9,402	Ontario-Born N= 37,608	PPs N= 14,255	Ontario-Born N= 57,020	NRIs N= 111,698	Ontario-Born N= 446,792
**Years 1 & 2**								
N with at least one minor illness	10, 260	36,678	7,885	27,291	11,585	42,729	8,3838	340,765
N (column %) excluded[a]	49 (0.0)	5,571 (0.11)	45 (0.0)	4,454 (0.12)	45 (0.0)	7,951 (0.14)	301 (0.0)	84,528 (0.19)
N (column %) with at least one minor illness after exclusions	10,211 (0.84)	31,107 (0.64)	7,810 (0.83)	23,467 (0.62)	11,540 (0.81)	34,778 (0.61)	83,537 (0.75)	256,237 (0.57)
**Primary outcome: All minor illnesses, seen in the ED**								
Total Population (N)	10,211	31,107	7,810	23,467	11,540	34,778	83,537	256,237
Minor ED visits, Mean (SD)	0.22 (0.66)	0.31 (0.71)	0.18 (0.50)	0.30 (0.67)	0.21 (0.54)	0.29 (0.66)	0.18 (0.51)	0.30 (0.66)
All Minor visits, Mean (SD)	5.47 (4.83)	3.88 (3.51)	4.76 (4.13)	3.89 (3.50)	4.37 (3.80)	3.92 (3.57)	4.39 (3.92)	4.13 (3.80)
**% of all minor illnesses seen in the ED**, **Mean (SD)**	6.06 (18.32)	9.92 (23.41)	5.09 (16.74)	9.87 (23.66)	6.02 (18.01)	9.36 (22.70)	5.00 (16.41)	9.45 (22.87)
**Sensitivity outcome: All minor illnesses, seen in the ED with a Family Practice Sensitive Condition (FPSC)**								
Minor ED visits with a FPSC, Mean (SD)	0.05 (0.29)	0.06 (0.33)	0.04 (0.24)	0.06 (0.30)	0.04 (0.25)	0.04 (0.26)	0.03 (0.24)	0.06 (0.30)
**% of all minor illnesses seen in the ED with a FPSC**, **Mean (SD)**	1.23 (9.16)	1.81 (10.97)	0.96 (7.65)	1.72 (10.98)	1.04 (8.02)	1.32 (9.48	0.82 (7.00)	1.55 (10.34)
**Complementary Measures**								
Routine PC visits, Mean (SD)	1.10 (1.34)	0.65 (0.95)	1.07 (1.27)	0.68 (0.97)	0.79 (1.07)	0.67 (0.95)	0.93 (1.14)	0.74 (0.99)
% with at least one routine PC visit (n)	56.3 (5,752)	43.1 (13,373)	57.7 (4,504)	45.2 (10,616)	48.0 (5,539)	45.2 (15,707)	54.9 (45,878)	48.5 (124,191)
Acute ED visits, Mean (SD)	0.33 (0.83)	0.37 (0.84)	0.30 (0.71)	0.39 (0.85)	0.28 (0.68)	0.32 (0.77)	0.25 (0.66)	0.37 (0.85)
**Years 3 & 4**								
Deaths in Y1/Y2 (column %)	1-5[b]	14	1-5[b]	11	1-5[b]	20	18	100
N with at least one minor illness	9,264	31,386	6,816	23,399	10,525	40,239	76,858	317,745
N (column %) excluded[a]	249 (0.0)	7,093 (0.15)	214 (0.0)	5,785 (0.15)	142 (0.0)	10,095 (0.18)	883 (0.0)	95,753 (0.21)
N (column %) with at least one minor illness after exclusions	9,015 (74.0)	24,293 (49.9)	6,602 (70.3)	17,614 (46.8)	10,383 (72.9)	30,144 (52.9)	75,975 (68.0)	221,992 (49.7)
**Primary outcome: All minor illnesses, seen in the ED**								
Total Population (N)	9,015	24,293	6,602	17,614	10,383	30,144	75,975	221,992
Minor ED visits, Mean (SD)	0.21 (0.57)	0.28 (0.65)	0.17 (0.47)	0.27 (0.63)	0.20 (0.53)	0.28 (0.69)	0.17 (0.49)	0.29 (0.66)
All Minor sick visits, Mean (SD)	4.41 (4.11)	3.30 (3.07)	3.81 (3.40)	3.29 (3.12)	3.82 (3.51)	3.75 (3.71)	3.95 (3.58)	3.83 (3.63)
**% of all minor sick visits seen in the E D, Mean (SD)**	6.72 (19.92)	10.42 (25.19)	6.35 (19.64)	10.12 (24.93)	6.74 (19.62)	9.65 (23.50)	5.36 (17.58)	9.49 (23.27)

© 2026 Wanigaratne S et al. *JAMA Network Open*.

Immigrant Groups and Ontario-born matches (1:4)	GARs N= 12,184	Ontario-Born N= 48,736	PSRs/BVORs N= 9,402	Ontario-Born N= 37,608	PPs N= 14,255	Ontario-Born N= 57,020	NRIs N= 111,698	Ontario-Born N= 446,792
**Sensitivity analysis: All minor illnesses, seen in the ED with a Family Practice Sensitive Condition (FPSC)**								
Minor ED visits with a FPSC, Mean (SD)	0.02 (0.17)	0.03 (0.21)	0.02 (0.15)	0.02 (0.20)	0.02 (0.15)	0.02 (0.17)	0.01 (0.15)	0.02 (0.20)
**% of all minor illnesses seen in the ED with a FPSC, Mean (SD)**	0.61 (6.98)	0.89 (7.87)	0.65 (7.66)	0.72 (6.82)	0.46 (5.11)	0.58 (6.09)	0.37 (5.01)	0.73 (7.24)
**Complementary Measures**								
Routine PC visits, Mean (SD)	0.73 (1.09)	0.57 (1.01)	0.63 (0.98)	0.61 (1.06)	0.66 (1.02)	0.71 (1.14)	0.77 (1.09)	0.71 (1.09)
% with at least one routine PC visit (n)	44.4 (4,000)	36.6 (8,901)	39.9 (2,633)	38.8 (6,843)	42.0 (4,364)	43.5 (13,115)	47.5 (36,092)	45.0 (99,975)
Acute ED visits, Mean (SD)	0.32 (0.81)	0.37 (0.89)	0.31 (0.76)	0.37 (0.85)	0.30 (0.74)	0.36 (0.89)	0.25 (0.68)	0.37 (0.89)

[a]primary analyses, immigrant children and youth with at least one minor sick visit were excluded if all their Ontario-born matches had no minor sick visits and all Ontario-born matches with at least one minor sick visit were excluded if their matched immigrant child had no minor sick visits. See eTable 12 for details.

[b]Small cells (<6) suppressed and non-missing data reported as ranges without percentage to reduce risk of re-identification in accordance with ICES policy.

© 2026 Wanigaratne S et al. *JAMA Network Open*.

eTable 11: Secondary outcomes among immigrant children and youth compared with their Ontario-born matches, with no minor illnesses (in either the emergency department or primary care), Years 1 and 2 and Years 3 and 4

Immigrant Groups and Ontario-born matches (1:4)	GARs N= 12,184	Ontario-Born N= 48,736	PSRs/BVORs N= 9,402	Ontario-Born N= 37,608	PPs N= 14,255	Ontario-Born N= 57,020	NRIs N= 111,698	Ontario-Born N= 446,792
**Years 1 & 2**								
N (column %), with no minor illnesses	1,924 (15.8)	12,058 (24.7)	1,547 (16.5)	9,687 (25.8)	2,670 (18.7)	14,291 (25.1)	27,860 (24.9)	106,027 (23.7)
Acute ED visits, Mean (SD)	0.15 (0.50)	0.13 (0.43)	0.13 (0.40)	0.15 (0.48)	0.11 (0.40)	0.12 (0.42)	0.06 (0.30)	0.14 (0.46)
Total Acute ED visits (N)	1-5[a]	19	1-5[a]	19	8	18	18	155
Routine primary care visits, Mean (SD)	0.63 (1.04)	0.41 (0.71)	0.69 (1.02)	0.45 (0.75)	0.50 (0.88)	0.48 (0.74)	0.42 (0.80)	0.51 (0.77)
**Years 3 & 4**								
N (column %), no minor illnesses	2,914-2918[b]	17,336 (35.6)	2,581-2585[b]	14,198 (37.8)	3,725-3729[b]	16,761 (29.4)	34,822 (31.2)	128,947 (28.9)
Acute ED visits, Mean (SD)	0.14 (0.51)	0.14 (0.45)	0.15 (0.46)	0.16 (0.48)	0.12 (0.43)	0.13 (0.43)	0.07 (0.31)	0.14 (0.46)
Total Acute ED visits (N)	7	44	1-5 [a]	29	1-5 [a]	27	31	188
Routine primary care visits, Mean (SD)	0.36 (0.74)	0.29 (0.62)	0.30 (0.64)	0.29 (0.61)	0.33 (0.69)	0.40 (0.74)	0.30 (0.68)	0.40 (0.72)

[a] Small cells (<6) suppressed and non-missing data reported as ranges without percentage to reduce risk of re-identification in accordance with ICES policy.
[b] Non-missing data reported as ranges without percentage to reduce risk of re-identification in accordance with ICES policy.

© 2026 Wanigaratne S et al. *JAMA Network Open*.

eTable 12: Frequencies for the top 5 *ICD-10* chapters for most responsible diagnosis for ED triage 4 to 5 visits for each exposure group and their matched group

Rank	Main diagnosis (ICD-10 CA)	Code description	Frequency	Rank	Main diagnosis (ICD-10 CA)	Code description	Frequency
**Government Assisted Refugees**				**Ontario-born Matches**			
**1**	J069	Acute upper respiratory infection, unspecified	205	**1**	J069	Acute upper respiratory infection, unspecified	802
**2**	B349	Viral infection, unspecified	158				
**3**	S0180	Open wounds of other parts of head, uncomplicated	127	**3**	S0180	Open wounds of other parts of head, uncomplicated	649
**4**	H669	Otitis media, unspecified	111	**2**	H669	Otitis media, unspecified	756
**5**	R104	Other and unspecified abdominal pain	98				
				**4**	S9349	Sprain and strain of ankle, unspecified	486
				**5**	S099	Unspecified injury of head	438
**Privately Sponsored Refugees/Blended Visa Office Referred Refugees**				**Ontario-born Matches**			
**1**	J069	Acute upper respiratory infection, unspecified	127	**1**	J069	Acute upper respiratory infection, unspecified	521
**2**	S0180	Open wounds of other parts of head, uncomplicated	92		S0180	Open wounds of other parts of head, uncomplicated	463
**3**	B349	Viral infection, unspecified	82	**3**			
**4**	H669	Otitis media, unspecified	69	**2**	H669	Otitis media, unspecified	476
**5**	R104	Other and unspecified abdominal pain	53				
				**4**	S9349	Sprain and strain of ankle, unspecified	386
				**5**	S099	Unspecified injury of head	309
**Successful Asylum Seekers (Protected Persons)**				**Ontario-born Matches**			
**1**	J069	Acute upper respiratory infection, unspecified	198	**2**	J069	Acute upper respiratory infection, unspecified	686
**2**	B349	Viral infection, unspecified	138				
**3**	S9349	Sprain and strain of ankle, unspecified	109	**1**	S9349	Sprain and strain of ankle, unspecified	784
**4**	H669	Otitis media, unspecified	108	**4**	H669	Otitis media, unspecified	573

© 2026 Wanigaratne S et al. *JAMA Network Open*.

Rank	Main diagnosis (ICD-10 CA)	Code description	Frequency	Rank	Main diagnosis (ICD-10 CA)	Code description	Frequency
**5**	R104	Other and unspecified abdominal pain	103				
				**3**	S0180	Open wounds of other parts of head, uncomplicated	636
				**5**	S099	Unspecified injury of head	515
**Non-refugee Immigrants**				**Ontario-born Matches**			
**1**	J069	Acute upper respiratory infection, unspecified	1268	**2**	J069	Acute upper respiratory infection, unspecified	6431
**2**	S0180	Open wounds of other parts of head, uncomplicated	1061	**1**	S0180	Open wounds of other parts of head, uncomplicated	6820
**3**	B349	Viral infection, unspecified	794				
**4**	H669	Otitis media, unspecified	787	**3**	H669	Otitis media, unspecified	6391
**5**	S9349	Sprain and strain of ankle, unspecified	687	**4**	S9349	Sprain and strain of ankle, unspecified	5258
				**5**	S099	Unspecified injury of head	4592

© 2026 Wanigaratne S et al. *JAMA Network Open*.

eTable 13: Frequencies for the top 5 *ICD-10* chapters for most responsible diagnosis for ED triage 4 to 5 visits with a family practice sensitive condition for each exposure group and their matched group

Rank	Main diagnosis (ICD-10 CA)	Code description	Frequency	Rank	Main diagnosis (ICD-10 CA)	Code description	Frequency
**Government Assisted Refugees (GARs**				**Ontario-born Matches**			
**1**	J069	Acute upper respiratory infection, unspecified	205	**1**	J069	Acute upper respiratory infection, unspecified	802
**2**	H669	Otitis media, unspecified	111	**2**	H669	Otitis media, unspecified	756
**3**	J029	Acute pharyngitis, unspecified	87	**3**	J029	Acute pharyngitis, unspecified	333
**4**	N390	Urinary tract infection, site not specified	35	**4**	N390	Urinary tract infection, site not specified	246
**5**	H109	Conjunctivitis, unspecified	18	**5**	H109	Conjunctivitis, unspecified	97
**Privately Sponsored Refugees/Blended Visa Office Referred Refugees (PSRs/BVORs)**				**Ontario-born Matches**			
**1**	J069	Acute upper respiratory infection, unspecified	127	**1**	J069	Acute upper respiratory infection, unspecified	521
**2**	H669	Otitis media, unspecified	69	**2**	H669	Otitis media, unspecified	476
**3**	J029	Acute pharyngitis, unspecified	38	**3**	J029	Acute pharyngitis, unspecified	239
**4**	H109	Conjunctivitis, unspecified	19	**5**	H109	Conjunctivitis, unspecified	59
**5**	N390	Urinary tract infection, site not specified	19	**4**	N390	Urinary tract infection, site not specified	164
**Successful Asylum Seekers or Protected Persons (PPs)**				**Ontario-born Matches**			
**1**	J069	Acute upper respiratory infection, unspecified	198	**1**	J069	Acute upper respiratory infection, unspecified	686
**2**	H669	Otitis media, unspecified	108	**2**	H669	Otitis media, unspecified	573
**3**	J029	Acute pharyngitis, unspecified	78	**3**	J029	Acute pharyngitis, unspecified	366
**4**	N390	Urinary tract infection, site not specified	39	**4**	N390	Urinary tract infection, site not specified	218
**5**	H109	Conjunctivitis, unspecified	30	**5**	H109	Conjunctivitis, unspecified	94
**Non-refugee Immigrants (NRIs)**				**Ontario-born Matches**			
**1**	J069	Acute upper respiratory infection, unspecified	1268	**1**	J069	Acute upper respiratory infection, unspecified	6431
**2**	H669	Otitis media, unspecified	787	**2**	H669	Otitis media, unspecified	6391
**3**	J029	Acute pharyngitis, unspecified	366	**3**	J029	Acute pharyngitis, unspecified	2744
**4**	N390	Urinary tract infection, site not specified	219	**4**	N390	Urinary tract infection, site not specified	2038
**5**	H109	Conjunctivitis, unspecified	176	**5**	H109	Conjunctivitis, unspecified	970

© 2026 Wanigaratne S et al. *JAMA Network Open*.

eReferences

1. Chiu M, Lebenbaum M, Lam K, et al. Describing the linkages of the immigration, refugees and citizenship Canada permanent resident data and vital statistics death registry to Ontario's administrative health database. *BMC medical informatics and decision making*. 2016;16:1-11.
2. Glazier RH, Klein-Geltink J, Kopp A, Sibley LM. Capitation and enhanced fee-for-service models for primary care reform: a population-based evaluation. *Cmaj*. 2009;180(11):E72-E81.
3. Weiner JP. The John Hopkins University Bloomberg School of Public Health, Health Services Research & Development Center. The John Hopkins ACG® Case-Mix System Version 6.0 Release Notes. 2003;
4. Matheson F. Moloney G (Unity Health Toronto), van Ingen T (Public Health Ontario). 2021 Ontario Marginalization Index: User Guide. *Toronto, ON: St Michael's Hospital (Unity Health Toronto) Joint Publication with Public Health Ontario*. 2023;
5. Matheson FOAfHPaPPHO. 2011 Ontario marginalization index: technical document. *Joint publication with Public Health Ontario*. 2017;(Toronto, ON: St. Michael's Hospital; 2017.)

© 2026 Wanigaratne S et al. *JAMA Network Open*.
